# Oxygen Minimum Zone and Organic Carbon Structure Benthic Prokaryotic Communities and Metabolism in Warm Deep-Red Sea Sediments

**DOI:** 10.3390/microorganisms14061191

**Published:** 2026-05-25

**Authors:** Wang Liu, Mongi Ennasri, Christopher A. Hempel, Mohammad A. Qurban, Carlos M. Duarte, Susana Agustí

**Affiliations:** 1 Biological and Environmental Sciences and Engineering Division, King Abdullah University of Science and Technology (KAUST), Thuwal 23955, Saudi Arabia; wang.liu@kaust.edu.sa (W.L.); mongi.ennasri@kaust.edu.sa (M.E.); chempel.work@gmail.com (C.A.H.); carlos.duarte@kaust.edu.sa (C.M.D.); 2 National Center for Wildlife (NCW), Riyadh 12411, Saudi Arabia; ceo@ncw.gov.sa

**Keywords:** Red Sea, oligotrophic, deep-sea sediments, oxygen minimum zone, benthic prokaryotes, bacterial community, carbon uptake, microbial networks, warm ocean

## Abstract

Benthic prokaryotic communities in deep-sea sediments remain poorly studied. They are constrained by organic matter availability and oxygenation in warm deep-sea ecosystems. Here, we investigated benthic prokaryotic communities and carbon uptake in deep Red Sea sediments (218–2415 m seafloor depth), where persistently warm (~21.5 °C) waters and a strong south–north productivity gradient co-occur. Sediment particulate organic carbon (POC), prokaryotic abundance (PA), and [^13^C]-D-glucose-based carbon uptake and uptake kinetics were examined in two sediment layers (0–1 and 4–5 cm), while bacterial communities were characterized using 16S rRNA gene sequencing of the 0–1 cm layer. Sediment POC, PA, and carbon uptake declined northward, consistent with reduced organic-carbon supply to the seafloor. Bacterial community composition differed significantly across the ~500 m depth associated with the Red Sea oxygen minimum zone (OMZ). Sediments from the relatively low-oxygen upper OMZ-range (200–500 m) had higher sediment POC and PA, and were enriched in putatively anaerobe-associated taxa, whereas deeper sediments (>500 m) below the OMZ exhibited more fragmented co-occurrence networks. These results suggest that organic-carbon availability defines the basin-scale metabolic backdrop, whereas bacterial community differentiation was more clearly resolved between upper OMZ-range and below-OMZ sediments than along latitude alone.

## 1. Introduction

The deep sea, typically defined as ocean regions below 200 m water depth, constitutes over 85% of the seafloor [1] and more than 60% of the Earth’s surface [2]. Yet it remains among the least characterized marine habitats [3]. As the terminal repository for exported organic matter, the seabed plays a central role in organic-matter degradation, remineralization, and long-term carbon burial [4,5]. Marine sediments also host one of the largest and most diverse microbial communities on Earth [6,7], but the spatial organization and environmental constraints of sedimentary microbial communities remain incompletely resolved [6,8], in part because deep-sea sampling is technically difficult and methodological heterogeneity complicates cross-study comparison [6].

Deep-sea benthic prokaryotic community composition and metabolic activity are shaped by multiple interacting controls, including sediment texture [9], substrate properties [3,7], porewater chemistry [3], redox gradients [10], organic-matter supply [3,7], oxygen availability [6,11], hydrostatic pressure [12,13], and hydrographic connectivity [8,14]. Collectively, these factors influence microbial colonization [15,16], carbon utilization [3], respiratory pathways [10], and the balance between biomass production and maintenance metabolism [17]. In addition, local substrate properties and geochemical context further shape benthic microbial assemblages: studies of shipwreck-associated sediments [18,19] and marine biofilms [20,21] have shown that benthic materials, contaminant exposure, source-water origin, substrate identity, and site conditions can modify microbial taxonomic composition and functional potential, including genes involved in sulfur, iron, and nitrogen cycling.

Among these interacting controls, organic-matter availability and oxygen supply are two main drivers of benthic microbial community composition and carbon cycling [6]. Organic matter availability regulates the energy and substrate supply that sustains microbial growth, carbon remineralization, and nutrient recycling in seafloor sediments [3,22]; oxygenation, in turn, governs the balance between aerobic remineralization and anaerobic pathways [11,23,24] that rely on alternative terminal electron acceptors and/or fermentation [25,26]. Dissolved oxygen (DO) in the deep sea is generally lower than in surface waters owing to biological consumption and reduced atmospheric exchange, but its concentration varies considerably across ocean basins depending on deep-water circulation and ventilation history [27,28]. The cold, recently ventilated waters of the Atlantic and Southern Ocean rank among the most oxygenated deep waters globally, whereas the Indian and Pacific Oceans maintain intermediate-to-low DO [27,29]. Because temperature directly affects oxygen solubility, anthropogenic warming is expected to reduce DO and impair deep-water ventilation, thereby intensifying ocean deoxygenation [30,31]. Such deoxygenation has significant consequences for deep-sea communities, including reduced aerobic metabolism, shifts in microbial and faunal composition, enhanced anaerobic processes, and altered carbon remineralization and nutrient cycling [27,32].

The Red Sea is a warm, oligotrophic basin with atypically warm deep waters (~21.5 °C) [33] that are currently undergoing rapid warming [34]. It is also characterized by relatively low but non-depleted oxygen concentrations, shaped by limited ventilation and high temperatures [35,36]. These coupled thermal, trophic, and oxygen characteristics make the basin well-suited for examining how organic-carbon limitation and relative oxygen depletion shape benthic microbial communities in a warm deep-sea basin. Surface productivity in the Red Sea is generally low but exhibits a robust south-to-north gradient, linked to monsoon-driven exchange of Indian Ocean intermediate water entering from the south through the Gulf of Aden [37,38,39]. Combined with the lack of riverine inputs and limited aeolian dust, this pelagic gradient establishes a corresponding gradient in organic carbon export that shapes deep-sea surface sedimentation, which is dominated by biogenic pelagic carbonates and contains only minor terrigenous material, mostly dust-derived [40,41,42]. In parallel, the basin’s semi-enclosed geometry and the narrow Bab el Mandeb exchange promote strong stratification and comparatively sluggish ventilation of intermediate-to-deep waters; together with heterotrophic oxygen consumption, these physical constraints give rise to the Red Sea oxygen minimum zone (OMZ) and drive spatial contrasts in bottom-water oxygenation [36,43,44]. Crucially, because oxygen solubility decreases with rising temperature, the Red Sea’s persistently warm deep waters are inherently predisposed to lower DO for a given ventilation rate, which can further reduce sedimentary oxygen exposure [45]. It should be noted that the Red Sea oxygen minimum is not a classical near-anoxic OMZ comparable to those of the eastern tropical Pacific, the eastern tropical Atlantic, or the northern Indian Ocean, including the Arabian Sea [25,46,47]. The depth of this relatively low-oxygen layer varies spatially and seasonally, with reported values ranging from approximately 325 to 570 m within the Red Sea water column [48]. Throughout this paper, the term “OMZ” refers to this relatively low-oxygen feature.

The Red Sea also contains a variety of deep-sea habitats, including hydrothermal vent fields and diffuse hydrothermal flow along the central ridge [49], as well as hypersaline anoxic brine pools (e.g., Atlantis II, Discovery, Shaban, and Kebrit) [50,51,52], all of which can host highly specialized microbial assemblages [49,50,51,52,53]. The present study, however, focuses on non-vent, non-brine seafloor habitats that dominate much of the basin and are expected to be shaped primarily by basin-scale gradients in particulate organic-carbon supply and bottom-water oxygenation.

In this study, we examined north–south variability in environmental parameters, prokaryotic standing stocks (prokaryotic abundance (PA) and prokaryotic biomass (PB)), [^13^C]-D-glucose-based carbon uptake, and 16S rRNA gene-based surface-sediment community composition in the warm deep sediments of the Eastern Red Sea basin. Our results show that sediment particulate organic carbon (POC), prokaryotic standing stocks, and bacterial carbon uptake all declined northward, consistent with basin-scale gradients in productivity and organic-carbon export. In addition, bacterial Shannon diversity and community composition differed significantly between sediments within the upper-OMZ range (200–500 m seafloor depth) and those in the below-OMZ (>500 m). The former were enriched in putatively anaerobe-associated families, including Anaerolineaceae, Desulfatiglandaceae, and Desulfosarcinaceae, whereas the latter displayed more fragmented co-occurrence networks. Seafloor depth and bottom-water DO emerged as the strongest predictors of bacterial community differentiation, indicating that bacterial composition differed more clearly between upper-OMZ range and below-OMZ sediments than along the basin-scale latitudinal organic carbon gradient alone.

## 2. Materials and Methods

### 2.1. Study Area and Sampling Design

**Figure 1:**
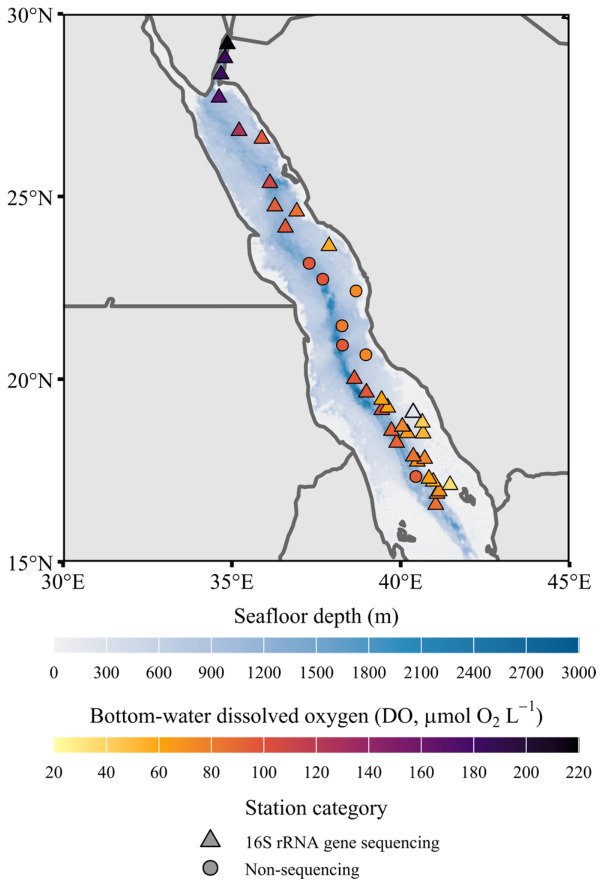
Sampling stations across the Eastern Red Sea basin overlaid on seafloor bathymetry (m). Symbols indicate station categories (triangles: 16S rRNA gene sequencing; circles: non-sequenced stations). The background color scale shows bathymetric depth, with darker blue indicating deeper areas. Point color denotes bottom-water dissolved oxygen (DO, μmol O_2_ L^−1^).

Sediment sampling was conducted aboard R/V OceanXplorer during the Red Sea Decade Expedition 2022 (4 February–15 June 2022). Sediment cores were collected at 39 stations distributed across the Eastern Red Sea basin (seafloor depths > 200 m), spanning a broad south-to-north gradient (16.56–29.17° N; Figure 1; Table S1). Coring was performed with acrylic push cores (10 cm internal diameter; mean penetration ~50 cm) deployed by an Argus Mariner XL Remotely Operated Vehicle (ROV). To minimize disturbance of the uppermost sediment centimeters, only cores with an intact sediment–water interface, clear overlying water, and no visible slumping or resuspension were retained for microbiological and biogeochemical analyses. When multiple intact cores were recovered at a station, a single core was selected for downstream processing.

Bottom water temperature and DO at each sediment-sampling station were recorded using a Sea-Bird SBE 911plus CTD mounted on the ROV; sensors had been calibrated by Sea-Bird Scientific (Bellevue, WA, USA) before the cruise. Water column DO profiles acquired from rosette-mounted CTD casts were used to place each sediment-sampling depth in the context of the water-column oxygen structure (Figure S1). A value of 62.5 μmol O_2_ L^−1^ (≈2 mg O_2_ L^−1^ [54]) was adopted as a reference hypoxia threshold. A 500 m seafloor depth was used to classify sediment location relative to the OMZ (Figure S1).

### 2.2. Sediment Collection and Processing

Two sediment layers (0–1 cm and 4–5 cm) were collected separately from each core. The sediment cores were sliced using a specialized core extruder (KC Denmark A/S, Silkeborg, Denmark), which enabled precise, high-resolution vertical sectioning at 0.5 cm intervals while minimizing disturbance and cross-contamination between layers. The 0–1 cm layer was sectioned first, immediately after core recovery, using sterile tools and the extruder. Grain-size distributions, sediment fabric, and porewater chemistry were not quantified, and visual heterogeneity was not systematically documented. We do not assume that community composition in the 4–5 cm layer matches that of the 0–1 cm layer. Comparisons with studies using thicker or homogenized sediment intervals are useful, but such studies address a different question from our surface-sediment comparison.

The two layers were used to quantify [^13^C]-D-glucose–based carbon-processing potential under standardized slurry incubations. Each layer was processed separately and homogenized with an equal volume of overlying bottom water using a vortex mixer (1 min) to produce independent sediment slurries (final slurry volume: 30–140 mL). Overlying water had been pre-filtered through 0.22-μm silver membrane filters (Sterlitech Corporation, Auburn, WA, USA). Slurry aliquots (1 mL; in replicates) were fixed with glutaraldehyde (1.0% final concentration) for prokaryotic abundance (PA) quantification and stored at −80 °C. The remaining slurry was allocated for measurement of the natural $\delta^{13}C$ of the prokaryotic fraction and for [^13^C]-D-glucose labeling assays (Sigma-Aldrich, Gillingham, Dorset, UK; CAS: 110187-42-3; Product No. 389374) [55] to determine bacterial production (BP) and uptake kinetics, as described below.

For community analysis, 16S rRNA gene sequencing was performed only on 0–1 cm surface sediments from 32 stations (Figure 1), because this layer represents the sediment-water interface most directly exposed to bottom-water oxygenation and recent organic-carbon deposition, and because molecular subsamples from the 4–5 cm layer were not preserved during the expedition. At each of these 32 stations, three biological subsamples (~5 g wet weight each) were collected from the same visually intact 0–1 cm layer with a sterile spatula [8]. Subsamples were transferred to sterile 15 mL centrifuge tubes, preserved with RNAlater (Thermo Fisher Scientific, Waltham, MA, USA) at a 1:1 volume-to-mass ratio, and stored at 4 °C until nucleic acid extraction. For the remaining seven stations included in the biogeochemical analyses, no corresponding sequencing data were available, as 0–1 cm sediment samples for molecular analysis were not collected.

The remaining sediment was retained at room temperature for subsequent sediment POC analysis.

### 2.3. Sediment Particulate Organic Carbon (POC)

Sediment samples were oven-dried (65 °C, 24 h) for dry weight determination (g); sediment volume (m^3^) was calculated from core geometry and was recorded only for areal or volumetric unit conversions, whereas POC was quantified on a dry-mass basis. Coarse fractions were pulverized using an agate mortar. Inorganic carbon was removed by treating 10.0 ± 0.1 mg aliquots of homogenized sediment in 10 mm × 10 mm silver capsules with 3 M HCl until reaction cessation. Acidified aliquots were redried (65 °C) and wrapped in tin capsules. Sediment POC content was quantified using a FLASH 2000 CHNS/O Elemental Analyzer (Thermo Scientific, Cambridge, UK), with acetanilide (71.09% C) and aspartic acid (36.09% C) as calibration and quality control standards, respectively.

### 2.4. Prokaryotic Standing Stocks (PA and PB)

To quantify prokaryotic standing stocks at each station, we measured prokaryotic abundance (PA, cells m^−2^) by flow cytometry [56]. A 50 mM sodium pyrophosphate (Na_4_P_2_O_7_) stock solution was added to thawed sediment slurries to a final concentration of 5 mM. After 15 min of dark incubation at room temperature, samples were vortexed (1 min) and sonicated (1 min) in an ice bath to prevent overheating. Prokaryotic cells were separated from sediment particles by centrifugation at 700× *g* (3000 rpm) for 5 min, yielding a supernatant containing the prokaryotic fraction [57]. Supernatants were stained with SYBR Green I (0.01% *v*/*v* final concentration) and incubated in the dark for 15 min. Prokaryotic cells were enumerated on a FACSCanto II flow cytometer (Becton Dickinson, Franklin Lakes, NJ, USA) [56]. Populations were gated based on green fluorescence (FITC-A; 530/30 nm), red fluorescence (PerCP-Cy5-A; 670/14 nm), and side scatter (SSC-A; 488 nm excitation). Counts were normalized on a volumetric basis by spiking with a calibrated fluorescent bead solution (1.21 × 10^6^ beads µL^−1^) [56]. The resulting counts represented the operationally defined prokaryotic-sized fraction recovered after sediment dispersion and low-speed centrifugation.

To estimate prokaryotic biomass (PB, g C m^−2^), a carbon per cell conversion factor of 2.2 × 10^−13^ g C μm^−3^ [58] and a mean cell volume of 0.1 μm^3^ cell^−1^ were used:

(1) $$PB=PA\times {2.2\times 10}^{-13}\times 0.1,$$

### 2.5. Natural $\delta^{13}C$, Carbon Uptake, and Specific Growth Rate

Heterotrophic carbon uptake was measured using [^13^C]-D-glucose as a tracer, following the stable isotope method described in Alothman et al. [55,59]. Organic carbon uptake is reported here as an estimate of bacterial production (BP), following Koshikawa et al. [60]. Carbon-uptake kinetics were estimated by exposing prokaryotic communities to a gradient of [^13^C]-D-glucose concentrations and fitting the resulting uptake responses to a Michaelis–Menten model. All [^13^C]-D-glucose incubations were conducted in sediment slurries at atmospheric pressure under standardized laboratory conditions. Reproducing in situ hydrostatic pressure on retrieved deep-sea sediments is technically demanding, and pressure can attenuate microbial activity [12,13]. Accordingly, BP, specific growth rate (SGR), and kinetic parameters were interpreted mainly as comparative measures among stations and sediment layers.

Replicate slurry aliquots were used to quantify the natural $\delta^{13}C$ of the prokaryotic fraction in each of the two sediment layers. Aliquots were processed directly by sonication (1 min) and centrifugation (176× *g*, 5 min) [57] to recover the prokaryotic fraction, after which supernatants were filtered through 0.22-μm silver membranes, foil-wrapped, and stored at −20 °C until analysis. In parallel, replicates for organic carbon uptake were amended with [^13^C]-D-glucose to a final concentration of 1867 nM and incubated for 24 h in the dark at in situ temperature with continuous gentle agitation (Hula Mixer, Thermo Fisher) before undergoing the same downstream processing.

Silver filters were acidified with 3 M HCl to remove inorganic carbon, dried, and analyzed for $\delta^{13}C$ and ^13^C enrichment using a combustion module (CM) attached to a cavity ring-down spectroscopy analyzer (CM-CRDS-G2201-i, Picarro, Santa Clara, CA, USA).

The fraction of ^13^C in each sample (F) was calculated from the isotope ratio (R) as described by Alothman et al. [55,59]:

(2) $$F=\frac{R}{R\;+\;1},$$

where R was derived from $\delta^{13}C$ (‰, relative to VPDB) as:

(3) $$R_{sample}=\;\left(\frac{\delta^{13}C}{1000}\;+\;1\right)\times \;R_{VPDB},$$

with $R_{VPDB}$ = 0.0114372 (Vienna Pee Dee Belemnite standard).

BP (mg C m^−2^ d^−1^) was calculated from the difference in ^13^C fraction between the enriched ($F_{samp}$) and natural ($F_{nat}$) samples:

(4) $$BP=\;\frac{\left(F_{samp}\;-\;F_{nat}\right)\;\times \;{POC}_{prok}}{s\;\times \;t},$$

where ${POC}_{prok}$ (g C) is the POC of the prokaryotic fraction collected on the filter; s (m^2^) is the sampled sediment surface area, calculated as the volume of dry sediment (m^3^) divided by the sampled-layer thickness (0.01 m); and t is the incubation time (d).

SGR (per day, d^−1^) was estimated by dividing BP by PB:

(5) $$SGR\;=\;\frac{BP}{PB},$$

Uptake kinetics were measured in replicated slurry aliquots in the 0–1 cm layer of four stations, amended with [^13^C]-D-glucose across a gradient of final concentrations (500, 1000, 1867, 2000, 3000, 4000, 5000, 7000, and 10,000 nM). At Station L2P1D7 (16.87° N), both the 0–1 cm and 4–5 cm sediment layers were incubated. Kinetic parameters were estimated by fitting uptake rates to a Michaelis–Menten equation:

(6) $$V\;=\;\frac{V_{max}\;\times \;S}{\left(K_{m}\;+\;S\right)},$$

where V is the uptake rate, $V_{max}$ is the maximum uptake rate at substrate saturation, S is the added-substrate concentration, and $K_{m}$ is the half-saturation constant (an apparent indicator of substrate affinity at the community level). The ratio $\frac{V_{max}}{K_{m}}$ was used as a community-level uptake efficiency index under low substrate concentrations.

### 2.6. Prokaryotic Community Composition (16S rRNA Gene)

#### 2.6.1. DNA Extraction, Amplification, and Sequencing

Genomic DNA was extracted independently from each of the three biological subsamples per station (300 mg sediment per extraction) using the DNeasy PowerSoil Pro Kit (Qiagen, Hilden, Germany) with laboratory-specific procedural adaptations to the manufacturer’s workflow [8]. Thus, each sequenced station was represented by three independent DNA extractions from three subsamples of the same 0–1 cm layer. Samples were homogenized with 800 µL CD1 buffer in PowerBead Pro tubes (Qiagen, Hilden, Germany) and incubated at 4 °C overnight. After incubation, samples were heated at 65 °C for 10 min and then mechanically lysed using a bead beater. After adding 600 µL CD3 buffer, samples were incubated on ice for 10 min, vortexed for 5 s, and centrifuged at 17,000× *g* for 5 min. DNA was eluted according to the manufacturer’s protocol. Final DNA concentrations were quantified using the Qubit dsDNA High Sensitivity Assay Kit on a Qubit 4.0 fluorometer (Thermo Fisher Scientific, Waltham, MA, USA).

The V3-V4 hypervariable region of the 16S rRNA gene was amplified using primers 341F and 805R [61]. Although these primers have been widely applied as “universal” prokaryotic primers, they were originally optimized for bacterial 16S templates and can exhibit multiple mismatches with archaeal sequences, potentially reducing archaeal amplification efficiency [62,63,64]. Accordingly, bacterial community patterns were treated as the primary community-composition results, whereas archaeal reads were retained as auxiliary information and interpreted conservatively at broad taxonomic levels rather than as a measure of absolute archaeal community coverage.

PCR reactions (25 µL total volume) contained: 5 µL KAPA HiFi HotStart ReadyMix (2×, Roche, Basel, Switzerland), 3.25 µL molecular-grade water, 1 µL each primer (5 µM, forward and reverse), 0.25 µL bovine serum albumin (20 mg mL^−1^, Thermo Fisher Scientific, Waltham, MA, USA), and 1 µL DNA template. Thermal cycling was carried out as follows: initial denaturation at 98 °C for 2 min; 28 cycles of 98 °C for 20 s, 54 °C for 20 s, and 72 °C for 15 s; followed by a final extension at 72 °C for 2 min. For each independent DNA extract, three technical-replicate PCR reactions were performed from the same DNA template and pooled to minimize stochastic amplification failures. Amplification success was verified by 1.5% agarose gel electrophoresis (100 V, 30 min) using a 100 bp DNA ladder.

Triplicate technical-replicate amplicons from each biological replicate were pooled in equimolar ratios and purified using AMPure XP magnetic beads (Beckman Coulter, Brea, CA, USA). Each of the three biological replicates per station was then processed and sequenced as an independent library, so that biological-replicate identity was preserved through DNA extraction, amplification, and sequencing. Sequencing libraries were constructed with the Illumina Nextera XT Library Preparation Kit (dual-indexed; Illumina, San Diego, CA, USA), followed by additional AMPure XP bead purification. Final libraries were quantified by Qubit and sequenced (2 × 250 bp paired-end) on an Illumina NovaSeq 6000 platform (Illumina, San Diego, CA, USA) at the King Abdullah University of Science and Technology (KAUST) Bioscience Core Lab.

#### 2.6.2. Bioinformatic Processing and Decontamination

Demultiplexing was carried out by the Bioscience Core Lab. Paired-end reads were processed with a modified version of APSCALE v1.6.3 [65], incorporating vsearch v2.20.0 [66], cutadapt v4.4 [67], and a Python 3.13.5 adaptation of LULU [68]. The workflow followed a “denoising-first” strategy to accommodate the high complexity of sediment datasets: raw reads were first quality-filtered by discarding sequences with >2 expected errors (maxEE = 2) and those outside the target amplicon length (350–550 bp). The filtered reads were then denoised with DnoisE v1.4.0 [69] to minimize sequencing-error–derived variants, and subsequently clustered with Swarm v3.1.4 [70] to generate denoised OTUs (dOTUs). To safeguard the biological integrity of the dataset, microDecon v1.0.2 [71] was applied to systematically remove reads detected in sequenced extraction blanks and PCR no-template controls. Default parameters were maintained for paired-end merging, dereplication, pooling, and LULU filtering. Although amplicon sequence variants (ASVs) are widely used in marker-gene studies [72], dOTUs were used here as a pragmatic unit for this highly diverse sediment dataset because denoising followed by fine-scale clustering and post-clustering curation can reduce error-derived noise and yield robust operational units for comparative community analyses [68,70]. This modified APSCALE pipeline and the associated wrapper scripts are available on GitHub (https://github.com/hempelc/apscale; https://github.com/hempelc/apscale_wrapper) (accessed on 11 November 2025).

#### 2.6.3. Taxonomic Assignment

Taxonomy was assigned to the resulting dOTUs using BLAST v2.16.0 [73] against the SILVA NR99 SSU Ref database (release 138.1 [72,74,75]) with an E-value threshold of 1 × 10^−5^. BLAST hits were further filtered as follows: hits with a bitscore below 150 and an alignment length below 100 bp were discarded. For each dOTU, only BLAST hits within the top 2% bitscore range were retained. Taxonomic assignment followed identity-based thresholds adapted from both classical [76] and recently updated boundaries [77] for prokaryotic taxonomy: species: ≥98%, genus: ≥95%, family: ≥90%, order: ≥85%, class: ≥80%, and phylum: ≥75%. Consensus taxonomy for each dOTU was then determined using the lowest common ancestor approach [78] applied to the retained BLAST hits.

### 2.7. Statistical Analyses and Visualization

All statistical analyses and visualizations of the 0–1 cm sediment community composition were conducted in R (v4.5.1). Read counts from the three independently extracted and sequenced biological-replicate libraries were first inspected at the station level and then summed to yield a single station-level dOTU table for downstream analyses, aligning the molecular dataset with the station-level biogeochemical measurements. Samples were grouped by seafloor depth relative to the OMZ, distinguishing the upper-OMZ range (200–500 m) from the below-OMZ (>500 m) sediments.

α diversity was quantified at each station using the Shannon index based on dOTU relative abundances. Differences in Shannon diversity between the two zones were evaluated with two-tailed Welch’s *t*-tests (*p* < 0.05) using complete cases.

β diversity patterns were evaluated using non-metric multidimensional scaling (NMDS) based on Bray–Curtis distance matrices. To down-weight rare taxa and improve robustness, ordinations and associated multivariate tests were performed using dOTUs collectively contributing to the top 90% of cumulative relative abundance within each domain (Figures S2 and S3). For environmental metadata, missing values were imputed using the median of the corresponding variable to retain stations in the ordinations. The significance of community separation between the two zones was evaluated using permutational multivariate analysis of variance (PERMANOVA). Environmental fitting was performed using candidate variables including bottom-water temperature, bottom-water dissolved oxygen (DO), sediment particulate organic carbon (POC), the natural $\delta^{13}C$ of the prokaryotic fraction, prokaryotic abundance (PA), and prokaryotic biomass (PB); only variables with significant fits were displayed in ordination figures. Differences in environmental and biological variables between the two zones were evaluated using two-tailed Welch’s *t*-tests (*p* < 0.05) with pairwise complete observations.

OMZ depth-discriminatory taxa were identified using a two-step procedure. First, taxa with significant differences in relative abundance between the upper-OMZ range and the below-OMZ were selected using two-sided Mann–Whitney U tests with Benjamini–Hochberg FDR correction (*q* < 0.05). Second, SIMPER analysis was performed to identify taxa that most strongly explained between-zone Bray–Curtis dissimilarity (i.e., taxa driving differences), and the top 20 contributors were retained. The selected taxa were therefore both statistically different between the two zones and among the disproportionate contributors to between-zone dissimilarity, rather than taxa defined by ubiquity/prevalence across zones. These taxa were visualized as violin plots (bacteria: family- and genus- levels; archaea: family level). Dataset-normalized relative abundance (read count per station divided by the total reads across the full dataset) was used rather than the station-wise relative abundance. For clarity, only the upper half of each violin was displayed, as relative abundance values are inherently non-negative.

To evaluate whether these OMZ depth-discriminatory taxa formed consistent, guild-like association patterns within each zone, correlation-based co-occurrence networks were constructed within each zone using the relative abundances of depth-discriminatory prokaryotic taxa at the phylum, class, and order levels. Pairwise correlations were estimated using Spearman rank correlations, and edges were retained where |*ρ*| ≥ 0.6 and the Benjamini–Hochberg FDR-adjusted *q* < 0.05. Networks were represented as weighted, undirected graphs and visualized with a force-directed layout. Because correlations in compositional amplicon data can reflect shared environmental responses and/or compositional constraints, these networks were interpreted as association structure rather than direct ecological interaction networks. Combined-domain (Bacteria + Archaea) networks were used to evaluate broad cross-domain co-variation, whereas bacterial-only and archaeal-only networks were provided as supplementary sensitivity analyses. Keystone taxa were operationally defined as nodes ranked within the top 20% for both degree and betweenness centrality.

## 3. Results

### 3.1. Environmental Gradients and Prokaryotic Standing Stocks

**Figure 2:**
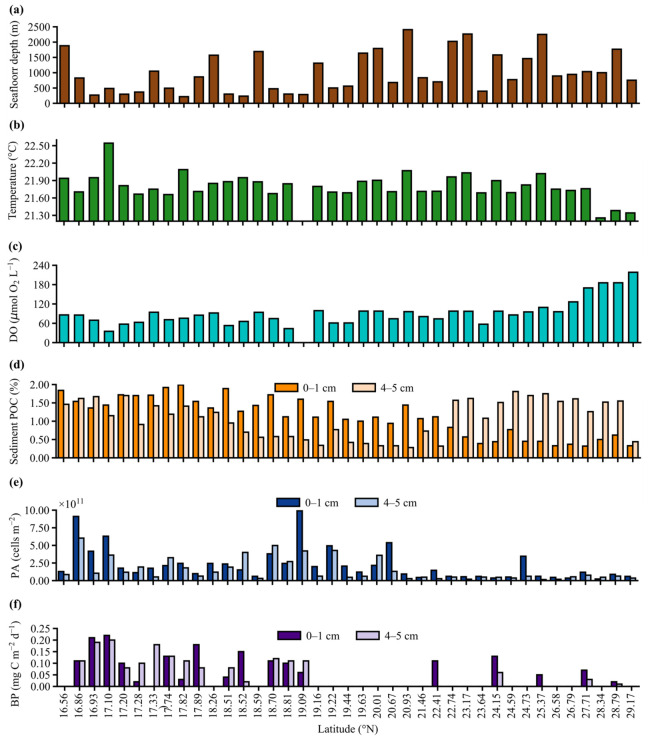
Geographical variability observed along the Eastern Red Sea (16.56–29.17° N). (**a**) Seafloor depth (m) of the sediments sampled; (**b**) bottom-water temperature (°C); (**c**) bottom-water dissolved oxygen (DO, μmol O_2_ L^−1^); (**d**) Sediment particulate organic carbon (POC, %); (**e**) prokaryotic cell abundance (PA, cells m^−2^), and (**f**) Bacterial production (BP, mg C m^−2^ d^−1^). Different color bars in (**d**–**f**) represent the two sediment layers (0–1 and 4–5 cm) analyzed. Missing bars indicate undetected values for the corresponding station and/or sediment layer.

Sediment samples were collected at seafloor depths ranging from 218 m to 2415 m, with the deepest sediments obtained from stations in the central basin (Figure 1 and Figure 2a; Table S1). By contrast, bottom-water temperatures were nearly isothermal, ranging from 21.26 °C to 22.54 °C (mean: 21.80 ± 0.22 °C; Table S1; Figure 2b). Bottom-water DO varied widely from 35.0 μmol O_2_ L^−1^ at Station L2P2D2 (17.10° N, 485 m seafloor depth) to 218.4 μmol O_2_ L^−1^ at Station L4P1D6 (29.17° N, 754 m), increasing northward with a mean of 92.3 ± 39.2 μmol O_2_ L^−1^ (Table S1; Figure S1; Figure 2c). Water-column DO profiles further showed that minimum DO values occurred mainly in the southern basin between 200 and 500 m depth, whereas deeper waters and northern stations were generally more oxygenated (Figure S1). This pattern indicated that most sediments sampled at seafloor depths shallower than 500 m across the sampled area were located within the Red Sea OMZ. Among stations with available bottom-water DO measurements, surface sediments collected in the upper-OMZ range (*n* = 12) had DO values below or only slightly above the reference hypoxia threshold, whereas those collected in the below-OMZ (*n* = 26) were almost entirely above this limit, with a single exception (61.0 μmol O_2_ L^−1^ at 562 m; Table S1; Figure S1). Spearman’s rank correlation further showed that DO increased significantly with both latitude (*ρ* = 0.64, *p* < 0.001) and seafloor depth (*ρ* = 0.78, *p* < 0.001).

Sediment POC did not differ significantly between 0–1 cm and 4–5 cm layers (*t* = 0.490, *p* = 0.625; Table S3), but the northward decline was visually clearer in the 0–1 cm layer than in the 4–5 cm layer (Figure 2d). Across these two layers, sediment POC ranged from 0.32% to 1.99%, with a mean of 1.10 ± 0.51%. Station-mean sediment POC values, calculated as the average of the two layers, were strongly negatively correlated with latitude (*ρ* = −0.89, *p* < 0.001) and decreased significantly with seafloor depth (*ρ* = −0.42, *p* < 0.01). Natural $\delta^{13}C$ values of the prokaryotic fraction across the two layers ranged from −59.96‰ at Station L4P1D7 (28.79° N, 1772 m) to −32.28‰ at Station L2P2D1 (16.93° N, 269 m), with a mean value of −47.24 ± 7.44‰ (Table S2), and did not differ significantly between layers (*t* = −0.181, *p* = 0.858; Table S3).

Across 0–1 cm and 4–5 cm layers, PA ranged from 1.54 × 10^10^ to 9.91 × 10^11^ cells m^−2^ (Table S2; Figure 2e), whereas PB varied between 3.39 × 10^−4^ and 2.18 × 10^−2^ g C m^−2^ (Table S2). Both variables showed a pronounced south–north decline, decreasing significantly with latitude (*ρ* = −0.64, *p* < 0.001) and seafloor depth (*ρ* = −0.54, *p* < 0.001). Neither PA nor PB differed significantly between the 0–1 cm and 4–5 cm layers (*t* = 1.585, *p* = 0.118; *n* = 39 for both; Table S3).

### 3.2. Bacterial Production and Uptake Kinetics Using [^13^C]-D-Glucose

Across 0–1 cm and 4–5 cm layers, BP varied from 0.01 to 0.22 mg C m^−2^ d^−1^ and was generally higher in the southern stations than in the northern ones, where it could not be quantified in a large number of stations because the natural isotopic signal was below the Picarro instrument’s reliable detection range (Table S2; Figure 2f). SGR ranged from 1.91 × 10^−3^ to 0.162 d^−1^ (Table S2). Neither BP nor SGR differed significantly between the 0–1 cm and 4–5 cm layers (BP: *t* = −0.041, *p* = 0.968; SGR: *t* = −0.316, *p* = 0.754; Table S3).

**Figure 3:**
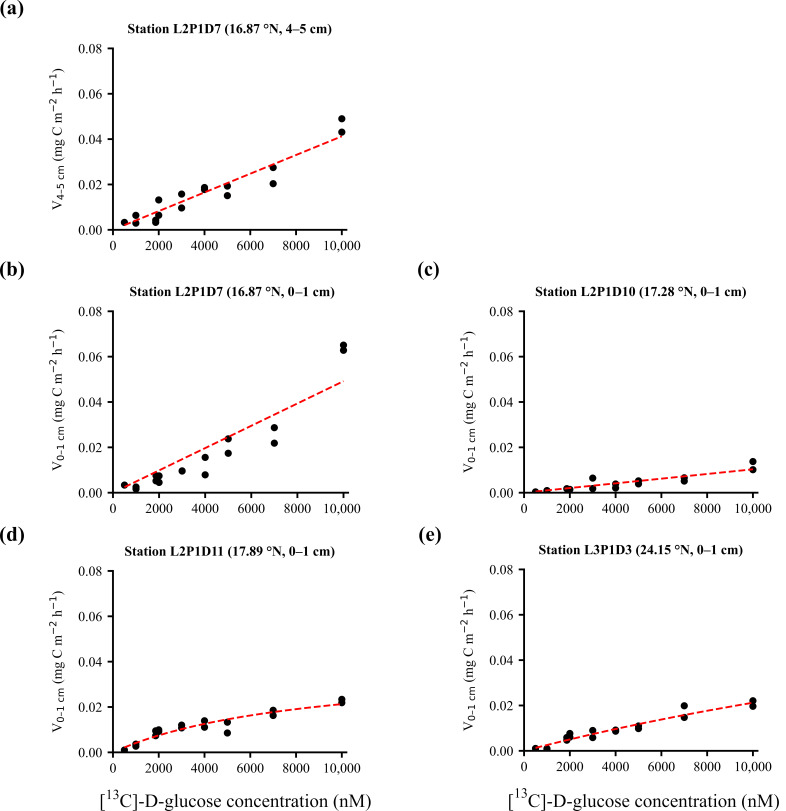
Michaelis–Menten curves of [^13^C]-D-glucose carbon uptake by prokaryotic communities from surface sediments at four deep Red Sea stations. (**a**) 4–5 cm and (**b**) 0–1 cm sediment layers from Station L2P1D7 (16.87° N). (**c**–**e**) 0–1 cm sediment layers from the other stations.

Michaelis–Menten kinetics of prokaryotic carbon uptake were significant in all five experiments (*p* < 0.001; Table 1) but showed substantial heterogeneity, with both linear and saturated responses observed (Figure 3a–e). Half-saturation constants ($K_{m}$) and maximum uptake rates ($V_{max}$) spanned up to four and three orders of magnitude, respectively. The highest values ($K_{m}$: 1.19 × 10^−2^ M; $V_{max}$: 58.37 mg C m^−2^ h^−1^) were observed at Station L2P1D7 (16.87° N), particularly in the 0–1 cm layer, whereas the lowest values ($K_{m}$: 8.53 × 10^−6^ M; $V_{max}$: 0.04 mg C m^−2^ h^−1^) were recorded at Station L2P1D11 (17.89° N). The uptake-efficiency index ($\frac{V_{max}}{K_{m}}$) varied from 1.03 × 10^3^ to 4.91 × 10^3^ mg C m^−2^ h^−1^ M^−1^ among curves, with the northernmost station yielding a relatively low value of 2.72 × 10^3^ mg C m^−2^ h^−1^ M^−1^ (Table 1).

### 3.3. Bacterial Community Composition (16S rRNA Gene)

Classified prokaryotic reads from the 0–1 cm sediment layer totaled 16,188,880, and yielded 189,636 dOTUs, of which 181,209 were assigned to Bacteria and 8427 to Archaea. Bacteria dominated read abundance (15,859,321 reads; 97.90%), whereas Archaea accounted for 323,015 reads (2.00%), and unidentified domain-level reads contributed the remaining 0.10%. Because the primer pair was expected to underrepresent Archaea, subsequent community interpretation focused on bacterial patterns, with archaeal results reported as auxiliary broad-scale information.

#### 3.3.1. Bacterial α Diversity (Shannon Index)

**Figure 4:**
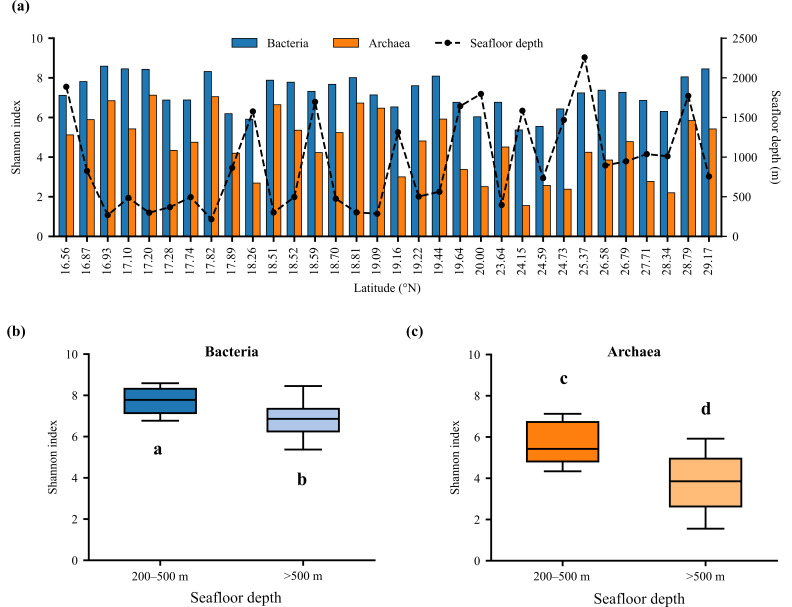
(**a**) Geographical distribution of the Shannon diversity index for bacterial (blue bars) and the identified archaeal (orange bars) taxa in the deep-sea surface sediments along the Eastern Red Sea, with seafloor depth overlaid as a dashed line. (**b**,**c**) Boxplots comparing Shannon diversity between the upper-OMZ range (200–500 m seafloor depth) and the below-OMZ (>500 m) for Bacteria (**b**) and Archaea (**c**). Boxes that do not share the same letter show significant differences (*p* < 0.05).

The bacterial Shannon index (Figure 4a) ranged from 5.37 at Station L3P1D3 (24.15° N, 1585 m seafloor depth) to 8.59 at Station L2P2D1 (16.93° N, 269 m), with a mean of 7.22 ± 0.88, indicating moderate-to-high within-station diversity. Bacterial diversity was significantly higher in the upper OMZ-range than in the below-OMZ (7.72 ± 0.64 vs. 6.88 ± 0.87, *t* = −3.154, *p* < 0.01; Figure 4b; Table 2) and decreased significantly with seafloor depth (*ρ* = −0.51, *p* < 0.01). The Shannon index for the identified archaeal taxa (Figure 4a,c) and the combined-domain Shannon index (Figure S4) showed similar depth-related patterns.

**Table 2:** Comparison of parameters between upper OMZ-range sediments (200–500 m) and below-OMZ sediments (>500 m) based on two-tailed Welch’s *t*-test (DO: dissolved oxygen; POC: particulate organic carbon; PA: prokaryotic abundance; PB: prokaryotic biomass; BP: bacterial production; SGR: specific growth rate). Statistically significant differences (*p* < 0.05) are shown in bold.

Parameters	Seafloor Depth	*n*	Mean ± SD	*t*	df	*p*
Shannon index-Bacteria	200–500 m	13	7.72 ± 0.64	−3.154	29.80	**<0.01**
	>500 m	19	6.88 ± 0.87			
Shannon index-Archaea	200–500 m	13	5.79 ± 1.04	−4.588	29.70	**<0.001**
	>500 m	19	3.82 ± 1.39			
Bottom-water temperature (°C)	200–500 m	12	21.87 ± 0.25	−1.249	17.51	0.228
	>500 m	19	21.77 ± 0.20			
Bottom-water DO (μmol O_2_ L^−1^)	200–500 m	12	60.5 ± 12.3	5.554	33.53	**<0.001**
	>500 m	19	107.0 ± 38.7			
Sediment POC (%)	200–500 m	13	1.26 ± 0.32	−2.364	24.04	**<0.05**
	>500 m	19	1.01 ± 0.29			
Natural $\delta^{13}C$ of prokaryotic fraction (‰)	200–500 m	10	−46.33 ± 7.22	−0.770	1.24	0.561
	>500 m	2	−51.77 ± 9.46			
PA (cells m^−2^)	200–500 m	13	(3.04 ± 1.76) × 10^11^	−3.155	20.99	**<0.01**
	>500 m	19	(1.23 ± 1.50) × 10^11^			
PB (g C m^−2^)	200–500 m	13	(6.67 ± 3.88) × 10^−3^	−3.155	20.99	**<0.01**
	>500 m	19	(2.72 ± 3.31) × 10^−3^			
BP (mg C m^−2^ d^−1^)	200–500 m	11	0.11 ± 0.05	−0.644	14.82	0.530
	>500 m	6	0.07 ± 0.05			
SGR (d^−1^)	200–500 m	11	(20.89 ± 11.88) × 10^−3^	1.808	7.50	0.111
	>500 m	6	(55.88 ± 53.81) × 10^−3^			

#### 3.3.2. Bacterial β Diversity (NMDS Ordination)

**Figure 5:**
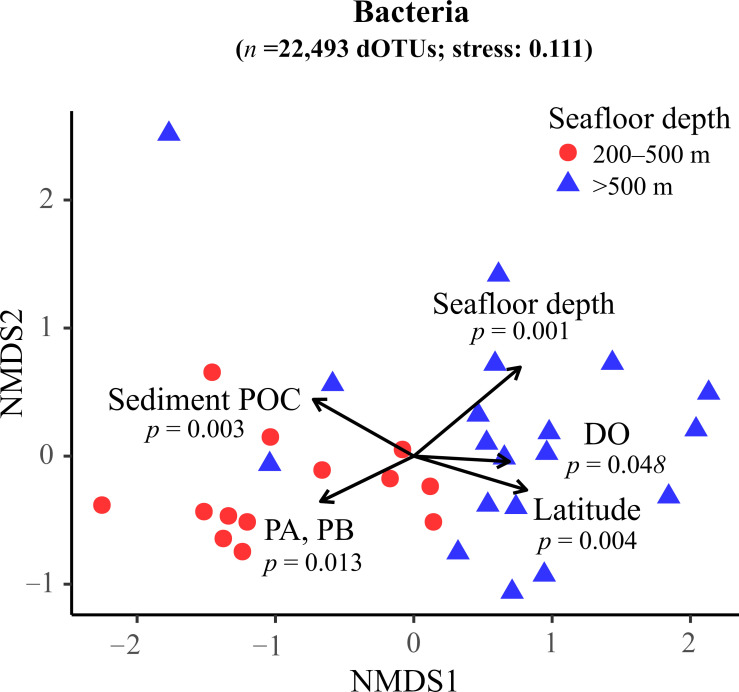
NMDS ordination (Bray–Curtis) of bacterial communities in the deep-sea surface sediments (0–1 cm below seafloor) along the Eastern Red Sea, based on the subset of dOTUs (denoised OTUs) cumulatively accounting for 90% of the total relative abundance, with benthic samples divided by their relation to the oxygen minimum zone (OMZ) (upper OMZ-range, red circles; below-OMZ, blue triangles). Black vectors show significant environmental fits (*p* < 0.05), including seafloor depth, bottom-water dissolved oxygen (DO), latitude, sediment particulate organic carbon (POC), prokaryotic abundance (PA), and prokaryotic biomass (PB); vector direction indicates increasing values, and vector length reflects the strength of correlation with the ordination.

NMDS ordinations of Bray–Curtis dissimilarities revealed clear separation of bacterial community composition between upper OMZ-range and below-OMZ sediments (Figure 5). Despite the lower abundance of archaeal reads, archaeal ordinations indicated a similar differentiation (Figure S5). Samples from the below-OMZ mainly clustered along the positive NMDS1 axis, whereas those from the upper-OMZ range clustered toward negative values along NMDS1, with limited overlap at the margins. Ordination fits were acceptable and reliable (bacteria: stress = 0.111; archaea: stress = 0.145). PERMANOVA confirmed that community composition differed significantly between the two sediment groups (*p* = 0.001).

Bacterial community composition was also related to environmental variability. Significant associations were detected with seafloor depth (R^2^ = 0.477, *p* = 0.001), latitude (R^2^ = 0.327, *p* = 0.004), and sediment POC (R^2^ = 0.321, *p* = 0.003), and weaker but still significant associations with prokaryotic standing stocks (R^2^ = 0.257, *p* = 0.013 for both PA and PB), and bottom-water DO (R^2^ = 0.197, *p* = 0.048), whereas it was independent of bottom-water temperature. Seafloor depth, latitude, and DO aligned with the positive NMDS1 axis, whereas sediment POC and prokaryotic standing stocks pointed in the opposite direction. Consistent with these NMDS patterns, Welch’s *t*-test supported significant differences between the upper-OMZ range and the below-OMZ for bottom-water DO (*t* = 5.554, *p* < 0.001), sediment POC (*t* = −2.364, *p* < 0.05), and prokaryotic standing stocks (*t* = −3.155, *p* < 0.01), whereas bottom-water temperature (*t* = −1.249, *p* = 0.228), natural $\delta^{13}C$ of the prokaryotic fraction (*t* = −0.770, *p* = 0.561), BP (*t* = −0.644, *p* = 0.530), and SGR (*t* = 1.808, *p* = 0.111) did not differ significantly (Table 2).

#### 3.3.3. Dominant Phylum-Level Prokaryotes

**Figure 6:**
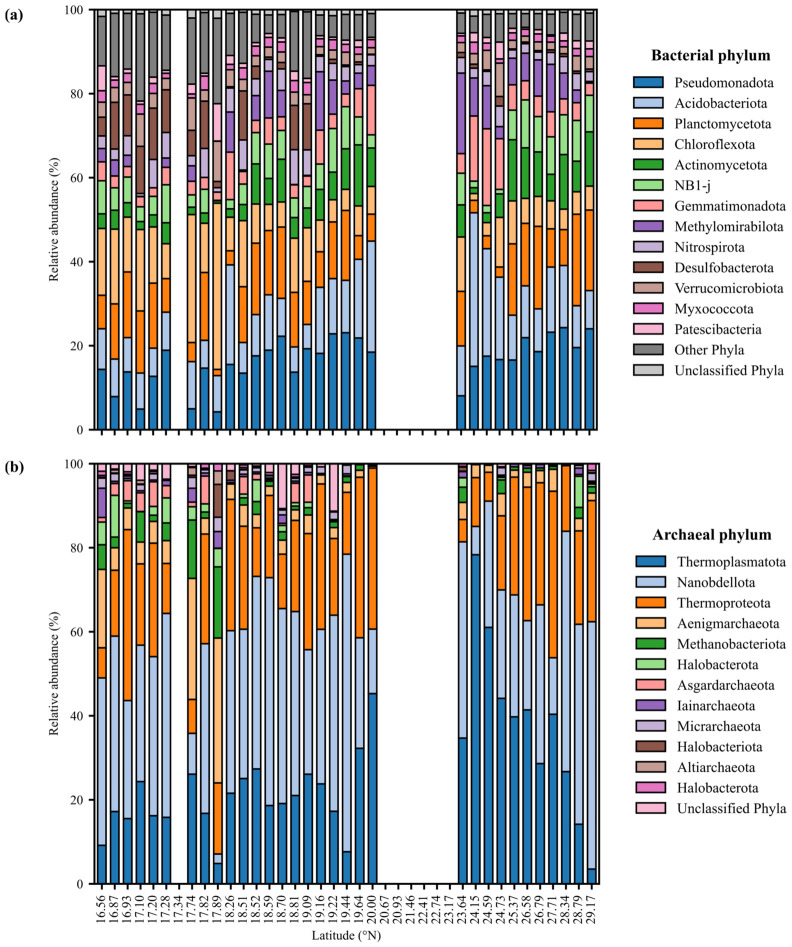
Latitudinal patterns in the relative abundance of dominant prokaryotic taxa in deep-sea surface sediments (0–1 cm below seafloor) along the Eastern Red Sea. Stacked bars show the community composition at the phylum level for Bacteria (**a**) and Archaea (**b**). The blank gap separates stations where 16S rRNA gene sequencing was unavailable.

Bacterial communities were dominated by 13 phyla that collectively accounted for 89.01% of reads (Figure 6a). Pseudomonadota was the most abundant phylum across nearly all stations. Acidobacteriota, Planctomycota, and Chloroflexota were also consistently abundant, while several additional phyla, including Methylomirabilota, Gemmatimonadota, and Nitrospirota, showed more patchy distributions. Importantly, phylum-level relative abundances differed significantly between upper OMZ-range and below-OMZ sediments (Table S4). Acidobacteriota (*t* = 2.701, *p* < 0.05), Actinomycota (*t* = 3.129, *p* < 0.01), Gemmatimonadota (*t* = 2.754, *p* < 0.05), and Methylomirabilota (*t* = 3.503, *p* < 0.01) were significantly lower in upper OMZ-range sediments than in below-OMZ sediments, whereas Chloroflexota (*t* = −2.218, *p* < 0.05) and Desulfobacterota (*t* = −2.842, *p* < 0.05) showed the opposite pattern. In addition, Pseudomonadota relative abundance also exhibited a clear latitudinal decline (*ρ* = −0.75, *p* < 0.001).

Although the primer set provided robust bacterial coverage, it also detected archaeal lineages assigned to 12 phyla (Figure 6b), with Thermoplasmatota, Nanobdellota, and Thermoproteota dominating the archaeal assemblages. Additional archaeal lineages included Aenigmarchaeota, Methanobacteriota, Iainarchaeota, Micrarchaeota, and Hadarchaeota, each contributing variably across stations. Between-zone archaeal differences were limited, with only Halobacteriota (*t* = −2.340, *p* < 0.05) and Asgardarchaeota (*t* = −2.743, *p* < 0.05) exhibiting markedly higher relative abundances in upper-OMZ range sediments than in the below-OMZ (Table S4).

#### 3.3.4. OMZ Depth-Discriminatory Bacterial Taxa

**Figure 7:**
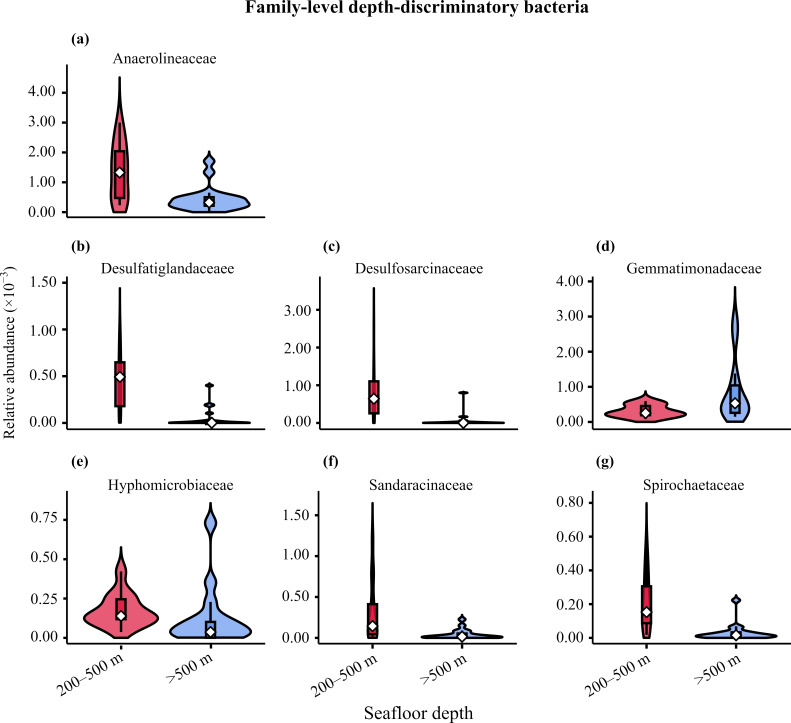
Family-level violin plots representing the relative reads of depth-discriminatory bacterial taxa from deep-sea surface sediments (0–1 cm below seafloor). Red and blue colors, as in Figure 5. (**a**) Anaerolineaceae. (**b**) Desulfatiglandaceae. (**c**) Desulfosarcinaceae. (**d**) Gemmatimonadaceae. (**e**) Hyphomicrobiaceae. (**f**) Sandaracinaceae. (**g**) Spirochaetaceae. Depth-discriminatory taxa were identified using a two-step selection: (i) taxa that differed significantly in relative abundance between the two depth zones (two-sided Mann–Whitney U test with Benjamini–Hochberg FDR-adjusted *q* < 0.05), and (ii) taxa ranked among the top 20 contributors to between-zone Bray–Curtis dissimilarity based on SIMPER analysis. The final set comprised taxa meeting both criteria. For clarity, only the upper half of each violin is shown.

**Figure 8:**
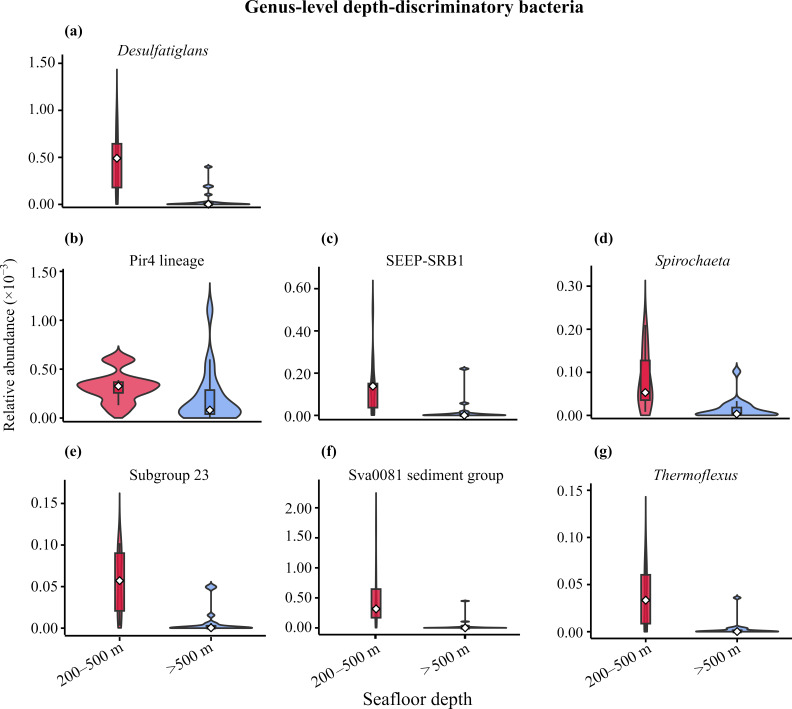
Genus-level violin plots representing the relative reads of depth-discriminatory bacterial taxa from deep-sea surface sediments (0–1 cm below seafloor). Red and blue colors as in Figure 7. (**a**) *Desulfatiglans*. (**b**) Pir4 lineage. (**c**) SEEP-SRB1. (**d**) *Spirochaeta*. (**e**) Subgroup 23. (**f**) Sva0081 sediment group. (**g**) *Thermoflexus*. Selection criteria as described in Figure 7. For clarity, only the upper half of each violin is shown.

The two-step procedure identified bacterial taxa with pronounced shifts that most strongly contributed to between-zone compositional separation (Figure 7 and Figure 8; Table S5; Figure S6). At the family level, upper OMZ-range sediments were enriched in Anaerolineaceae (Benjamini–Hochberg FDR–corrected *q* < 0.01), Desulfatiglandaceae (*q* < 0.001), Desulfosarcinaceae (*q* < 0.001), Hyphomicrobiaceae (*q* < 0.01), Sandaracinaceae (*q* < 0.01), and Spirochaetaceae (*q* < 0.001), whereas Gemmatimonadaceae (*q* < 0.05) was more abundant in the below-OMZ sediments (Figure 7a–g; Table S5).

At the genus level (Figure 8a–g; Table S5), several depth-discriminatory bacterial genera showed significantly higher relative abundances in upper OMZ-range sediments, including *Desulfatiglans* (*q* < 0.001), Pir4 lineage (*q* < 0.05), SEEP-SRB1 (*q* < 0.001), *Spirochaeta* (*q* < 0.001), Subgroup 23 (*q* < 0.01), Sva0081 sediment group (*q* < 0.001), and *Thermoflexus* (*q* < 0.001).

#### 3.3.5. Prokaryotic Co-Occurrence Networks

**Figure 9:**
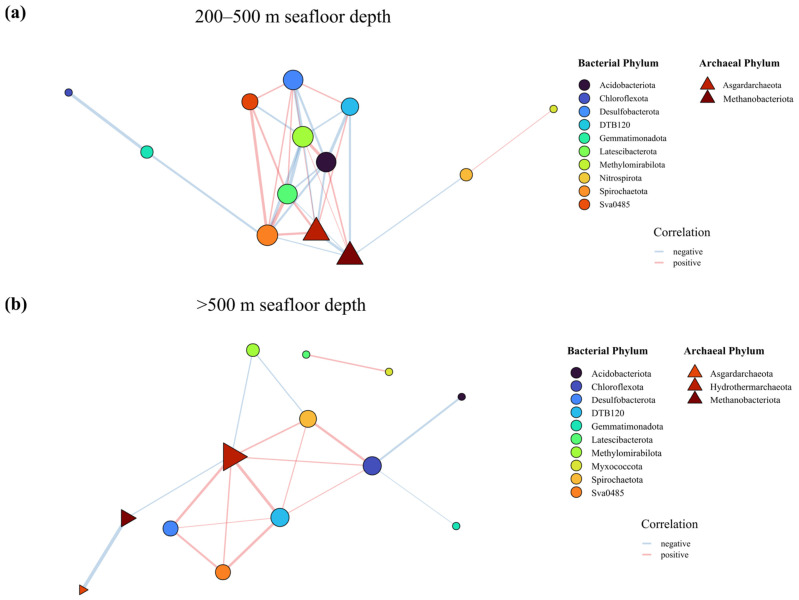
Co-occurrence networks of benthic combined-domain (Bacteria + Archaea) communities at the phylum level in deep-sea surface sediments (0–1 cm below seafloor) constructed from Spearman correlations of relative abundances. (**a**,**b**) represent communities at 200–500 m and >500 m seafloor depths, respectively. Depth-discriminatory taxa were identified using a two-step selection: (i) taxa that differed significantly in relative abundance between the two zones (two-sided Mann–Whitney U test with Benjamini–Hochberg FDR-adjusted *q* < 0.05), and (ii) taxa ranked among the top 20 contributors to between-zone Bray–Curtis dissimilarity based on SIMPER analysis. The final set comprised taxa meeting both criteria. Edges were retained when |*ρ*| ≥ 0.6 and Benjamini–Hochberg FDR-adjusted *q* < 0.05; red edges indicate positive correlations and blue edges indicate negative correlations, with edge width proportional to |*ρ*|. Nodes represent depth-discriminatory taxa; node size is proportional to mean relative abundance within each zone, and node color distinguishes individual taxa.

**Figure 10:**
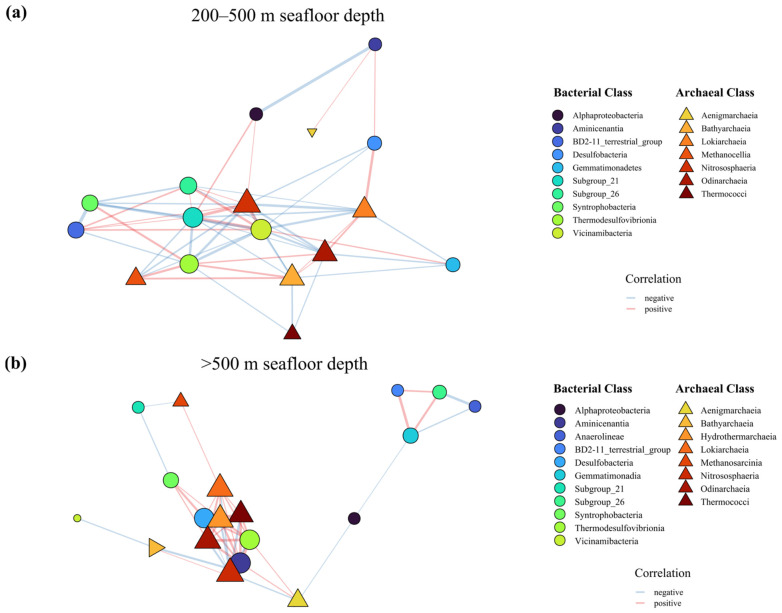
Co-occurrence networks of benthic combined-domain (Bacteria + Archaea) communities at the class level in deep-sea surface sediments (0–1 cm below seafloor) constructed from Spearman correlations of relative abundances. Selection criteria as described in Figure 9. (**a**,**b**) represent communities at 200–500 m and >500 m seafloor depths, respectively. Edges and nodes as described in Figure 9.

**Figure 11:**
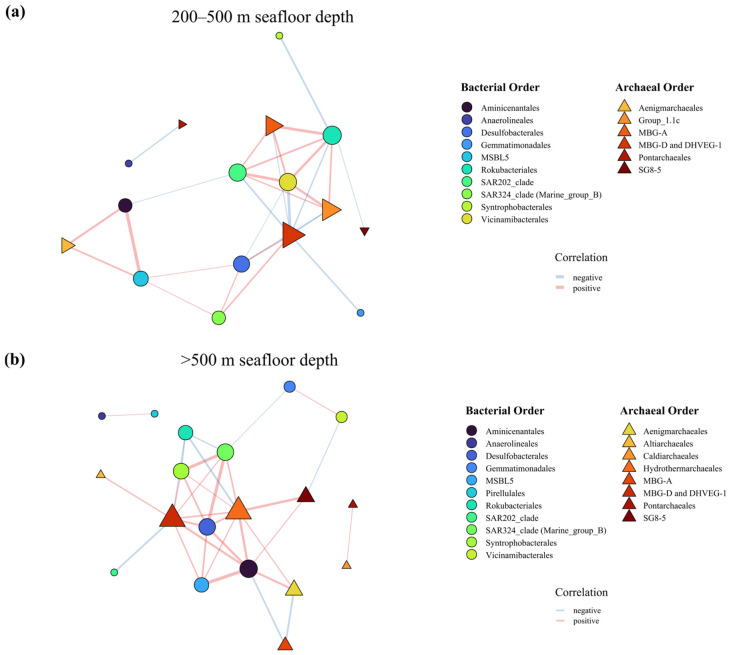
Co-occurrence networks of benthic combined-domain (Bacteria + Archaea) communities at the order level in deep-sea surface sediments (0–1 cm below seafloor) constructed from Spearman correlations of relative abundances. Selection criteria as described in Figure 9 and Figure 10. (**a**,**b**) represent communities at 200–500 m and >500 m seafloor depths, respectively. Edges and nodes as described in Figure 9 and Figure 10. MBG-D and DHVEG-1: Marine Benthic Group D and Deep-sea Hydrothermal Vent Methanobacterial Group 1. MBG-A: Marine Benthic Group A.

Co-occurrence networks of depth-discriminatory taxa displayed distinct topologies between zones. In the combined-domain (Bacteria + Archaea) networks, upper-OMZ range communities were better connected (i.e., fewer disconnected components), whereas the communities in the below-OMZ sediments were more fragmented (Figure 9, Figure 10 and Figure 11; Tables S6 and S7). The same broad contrast was also evident in the bacterial-only networks, supporting the bacterial community patterns identified by ordination and differential-abundance analyses (Figures S7–S9; Table S8).

At the phylum level, the upper-OMZ range network was centered on Spirochaetota (Bacteria; motile spirochetes commonly reported in aquatic environments and surface sediments), which formed one of the strongest retained positive links with Desulfobacterota (ρ = 0.703, FDR-adjusted *q* < 0.05) (Figure 9a; Table S7). In the below-OMZ network, keystone positions shifted to Chloroflexota (Bacteria) and Hydrothermarchaeota (Archaea) (Figure 9b; Table S7). Chloroflexota retained a strong positive association with Spirochaetota (ρ = 0.774, *q* < 0.01), whereas Hydrothermarchaeota was tightly linked with Desulfobacterota (ρ = 0.782, *q* < 0.01) and Spirochaetota (ρ = 0.711, *q* < 0.01).

At the class level, the upper-OMZ range network was organized around two keystone classes, Vicinamibacteria (Bacteria) and Nitrososphaeria (largely ammonia-oxidizing archaea; key marine nitrifiers) (Figure 10a; Table S7). Both keystones retained strong negative associations with classes such as Thermodesulfovibrionia (including sulfate reducers) and Desulfobacteria (all *q* < 0.01). In the below-OMZ network, no keystone was identified. However, Nitrososphaeria retained prominent negative links with Thermodesulfovibrionia (ρ = −0.800, *q* < 0.001), Desulfobacteria (ρ = −0.604, *q* < 0.05), and Aminicenantia (an iron-reduction–associated lineage; ρ = −0.847, *q* < 0.001), even though the retained edges were dominated by overall positive associations (Figure 10b; Table S7).

At the order level, keystone positions in the upper OMZ-range network were occupied by the bacterial orders Rokubacteriales and SAR202_clade (Chloroflexota) together with the archaeal lineages MBG-D and DHVEG-1 (Marine Benthic Group D and Deep-sea Hydrothermal Vent Methanobacterial Group 1) (Figure 11a; Table S7). The strongest retained associations highlighted tight co-variation among bacterial orders (e.g., SAR202_clade–Vicinamibacterales, *ρ* = 0.901, *q* < 0.01). In the below-OMZ network, keystone positions shifted toward Aminicenantales (Bacteria) and Hydrothermarchaeales (Archaea), while MBG-D and DHVEG-1 remained keystone taxa (Figure 11b; Table S7). Here, Desulfobacterales co-varied strongly and positively with Aminicenantales (*ρ* = 0.789, *q* < 0.01) and Hydrothermarchaeales (*ρ* = 0.719, *q* < 0.01).

## 4. Discussion

Our results indicated that organic-carbon availability and the Red Sea OMZ jointly structured benthic prokaryotic communities and metabolism in the deep-sea sediments.

Sediment POC, prokaryotic standing stocks (PA, PB), and bacterial carbon uptake declined from south to north, consistent with the spatial pattern in organic-carbon supply to the seafloor and providing the primary basin-scale ecological backdrop for benthic prokaryotic communities in surface sediments. This pattern agrees with the oligotrophic nature of the Red Sea and its well-established northward decline in pelagic productivity and nutrient availability: the northern basin is generally more nutrient-depleted and less productive than the southern Red Sea, where nutrient-rich water intrusions from the Gulf of Aden enhance production [37,79,80]. Furthermore, the export of fresh organic matter to the deep seafloor is constrained by the absence of substantial riverine inputs and by intense remineralization during particle sinking, so that only a small fraction of exported POC reaches depth [37,81]. In this context, the frequent inability to quantify bacterial carbon uptake at northern stations further underscored the consequences of reduced downward carbon delivery and stronger carbon limitation at the seabed. Although hydrostatic pressure has been shown to suppress prokaryotic carbon conversion efficiency under in situ conditions [12,13], atmospheric-pressure incubations remain the standard approach for carbon-uptake measurements in deep-sea studies. Thus, BP and uptake-kinetic values reported here should be interpreted as standardized comparative responses rather than absolute in situ rates.

The strongly negative natural $\delta^{13}C$ values of the prokaryotic fraction (down to ~−60‰; Table S2) can also be interpreted in the context of these carbon-poor and relatively low-oxygen deep sediments. In oligotrophic oceans, freshly exported phytoplankton-derived material is progressively consumed, reworked, and mixed with older or more degraded organic matter during sinking and after deposition, so that the organic matter remaining in surface sediments does not necessarily retain the isotopic signature of fresh pelagic production [82,83]. Therefore, strongly depleted microbial $\delta^{13}C$ values could reflect the incorporation of highly reworked and isotopically altered organic carbon under carbon-limited sediment conditions. Alternatively, they may indicate contributions from ^13^C-depleted methane-related carbon pools. However, because methane concentrations, methane-cycling functional genes (e.g., *mcrA* and *pmoA*), and porewater geochemistry were not measured, this possibility remains speculative.

Superimposed on this basin-scale carbon-supply gradient was bottom-water oxygenation associated with the depth range of the OMZ. The Red Sea oxygen minimum is not a classical near-anoxic OMZ comparable to those of the eastern tropical Pacific, the eastern tropical Atlantic, or the northern Indian Ocean [25,46,47], but this oxygenation pattern is particularly relevant in this basin because its deep water is exceptionally warm for the deep ocean (~21.5 °C) [33], in contrast to the ~4 °C typical of most other basins. The unusually high temperature lowers oxygen solubility and, together with restricted deep-water renewal across the Bab el Mandeb, helps maintain relatively low DO concentrations compared with most other deep-sea settings [33,44]. NMDS ordination, environmental fitting, and depth-group comparisons revealed that bacterial community differentiation in surface sediments was more clearly resolved between upper-OMZ range and below-OMZ sediments than along latitude alone (Figure 5; Figure S5). Specifically, upper OMZ-range sediments (200–500 m seafloor depth) were more closely aligned with higher organic-matter supply and prokaryotic standing stocks, whereas below-OMZ sediments (>500 m) were more closely associated with higher bottom-water DO.

Consistent with the differential-abundance analyses, surface sediments within the upper OMZ range showed higher relative abundances of bacterial families such as Anaerolineaceae, Desulfatiglandaceae, and Desulfosarcinaceae (Figure 7a–g), whose members have been reported to be involved in fermentative and sulfur-reducing pathways [84,85,86]. Notably, some Anaerolineaceae genomes have been reported to encode potential nitrate/nitrite-reduction pathways that would be constrained where nitrate is scarce [86,87], as in the oligotrophic Red Sea. Analogously, under sulfate-limited conditions, certain Desulfobacterota have been reported to shift away from canonical sulfate reduction and to rely more on fermentative and/or syntrophic metabolism [88,89]. By contrast, Gemmatimonadaceae was the only family consistently more abundant in below-OMZ sediments. Members of this lineage are commonly found across diverse habitats (including soils, aquatic environments, and engineered systems such as wastewater treatment) [90,91,92]. Published genomic and physiological studies have described traits linked to the utilization of complex organic compounds [93,94], a niche broadly compatible with relatively more oxygenated, low-POC surface sediments.

Co-occurrence network analyses provided further insight into the community differentiation between upper OMZ-range and below-OMZ sediments. The 341F/805R primer pair used here likely underrepresented archaeal templates [62,63,64], so archaeal signals are interpreted as auxiliary information on archaeal community structure. Within this limit, network topology primarily reflected habitat filtering [95] driven by the combined effects of bottom-water oxygenation and organic-matter availability. Upper OMZ-range communities showed stronger connectivity, consistent with more cohesive co-variation among taxa sharing relatively low-oxygen and higher-POC niches [96], whereas greater fragmentation characterized below-OMZ communities. At the phylum level, the upper OMZ-range network was organized around Spirochaetota and retained a strong positive association with Desulfobacterota, supporting co-variation among lineages that commonly become more abundant under oxygen-deficient conditions (Figure 9a). In contrast, the below-OMZ phylum-level network shifted keystone positions toward Chloroflexota and Hydrothermarchaeota, with Hydrothermarchaeota showing tight positive links to Desulfobacterota and Spirochaetota (Figure 9b), suggesting proportionally stronger cross-domain co-variation (i.e., concordant niche sorting) despite lower overall network cohesion. At the class level, Vicinamibacteria and Nitrososphaeria structured the upper OMZ-range network, whereas no keystone was identified in the below-OMZ network, although Nitrososphaeria retained strong negative links to Thermodesulfovibrionia, Desulfobacteria, and Aminicenantia, consistent with a recurrent gradient contrasting predominantly ammonia-oxidizing lineages with taxa more often reported from low-oxygen or reduced sediment settings [97,98,99]. Order-level networks further refined this pattern by showing increased fragmentation at finer taxonomic resolution, especially in the below-OMZ (three components vs. two in the upper OMZ-range) (Figure 11).

The community patterns documented above may also have been modulated by sediment chemical properties. Analyses of Red Sea sediment cores spanning 500 years by Cai et al. [100] revealed declines in element accumulation rates for nitrogen (−14.0%), phosphorus (−16.4%), and sulfur (−17.2%) in the southern basin—consistent with reduced Indian Ocean nutrient inflow driven by warming-induced stratification—and increasing accumulation rates of trace metals (iron, cadmium, vanadium, zinc, copper, chromium, and nickel) in the northern basin, linked to anthropogenic inputs following the opening of the Suez Canal. Although these chemical properties were not measured in the present study, their basin-scale co-variation with organic carbon may contribute to additional structuring of benthic prokaryotic communities beyond the carbon and oxygenation effects identified here. Furthermore, while our sampling avoided known brine pools, the Red Sea rift hosts widespread diffuse hydrothermal activity [49] that may locally enrich sediment pore waters in metals and reduced sulfur species, potentially creating microhabitats for specialized taxa. Future studies integrating porewater geochemistry and sediment elemental analyses would help disentangle these contributions.

The 0–1 cm and 4–5 cm sediment layers showed no statistically significant differences in sediment POC, PA, PB, BP, or SGR (Table S3), consistent with limited recent changes in sediment accumulation inferred from ^210^Pb chronologies in upper Red Sea sediment cores [100], as well as the slow sedimentation rates and limited bioturbation characteristic of oligotrophic deep-sea settings [101,102]. Community composition analysis, therefore, focused on the 0–1 cm layer, which represents the most biogeochemically active interface with the overlying water column. Nevertheless, the absence of community data from the 4–5 cm layer precluded direct assessment of vertical shifts in prokaryotic assemblages between these horizons. In other deep-sea regions, substantial vertical community turnover has been documented over centimeter-to-decimeter depth intervals, driven primarily by redox zonation and shifts in organic substrate quality [103,104,105,106,107]. In the Red Sea specifically, measurements in the Gulf of Aqaba have shown oxygen penetration depths ranging from 2 to 21 mm, depending on sediment grain size and organic loading [108], implying that steep redox gradients develop within the uppermost centimeters. Thus, the extent to which such vertical stratification occurs in these oligotrophic Red Sea sediments merits future investigation through high-resolution core profiling.

In terms of broader research context, this study, together with the concurrent work of Hempel et al. [8], constitutes the first comprehensive characterization of benthic prokaryotic communities across a large spatial domain of the deep Red Sea basin. Prior microbial studies in the Red Sea were largely restricted to highly specialized brine-pool and hydrothermal-vent environments [109,110,111] or focused on coastal habitats [112]. Hempel et al. [8] examined benthic prokaryotic communities across the Red Sea’s latitudinal gradient and three depth strata, finding clear biogeographic patterns shaped by depth, latitude, and oxygen availability—results broadly consistent with the community differentiation reported here.

## 5. Conclusions

Across 39 stations in Eastern Red Sea deep-sea sediments, organic-carbon availability and bottom-water DO played complementary ecological roles in benthic bacterial communities and carbon uptake. The overall northward declines in sediment POC, prokaryotic standing stocks (PA, PB), and bacterial carbon uptake (BP and uptake kinetics) reflected a basin-scale gradient in organic-carbon supply. By contrast, bacterial community composition and co-occurrence structure were more clearly differentiated between upper OMZ-range and below-OMZ sediments than along the broad latitudinal productivity gradient alone. Specifically, upper OMZ-range sediments were enriched in putatively anaerobe-associated bacterial taxa, whereas below-OMZ sediments exhibited more fragmented co-occurrence networks.

This study provides a basin-scale reference for understanding how organic-carbon limitation and bottom-water DO conditions shape benthic microbial ecology in a warm deep-sea basin. More integrative studies combining biogeochemical measurements, high-resolution sediment-core analyses, and genome-resolved approaches, including metagenomics and metatranscriptomics, will help clarify the bacterial metabolisms, carbon-transformation pathways, and redox-linked microbial functions that dominate warm, oligotrophic deep benthic environments, and assess how these communities may respond to the projected intensification of ocean warming and deoxygenation.

## Figures and Tables

**Table 1:** Michaelis–Menten kinetics of [^13^C]-D-glucose carbon uptake measured from 5 deep-sea benthic community incubations. All regressions were significant at *p* < 0.001.

Station	Latitude (° N)	Depth of Sediment Cores (m)	Sediment Layer	$K_{m}$(M)	V_max_ (mg C m^−2^ h^−1^)	R^2^	$\frac{V_{max}}{K_{m}}$(mg C m^−2^ h^−1^ M^−1^)
L2P1D7	16.87	826	4–5 cm	5.92 × 10^−3^	24.46	0.908	4.13 × 10^3^
L2P1D7	16.87	826	0–1 cm	1.19 × 10^−2^	58.37	0.837	4.91 × 10^3^
L2P1D10	17.28	368	0–1 cm	2.96 × 10^−3^	3.06	0.830	1.03 × 10^3^
L2P1D11	17.89	864	0–1 cm	8.53 × 10^−6^	0.04	0.884	4.69 × 10^3^
L3P1D3	24.15	1585	0–1 cm	4.05 × 10^−5^	0.11	0.931	2.72 × 10^3^

## Data Availability

The original contributions presented in the study are included in the article and Supplementary Material. Sequence data are openly available in BioProject PRJNA1216728 at https://www.ncbi.nlm.nih.gov/sra (accessed on 11 November 2025).

## Supplementary Materials

The following supporting information can be downloaded at: https://www.mdpi.com/article/10.3390/microorganisms14061191/s1, Table S1: Seafloor conditions along the Eastern Red Sea; Table S2: Seafloor conditions and sediment microbial metrics across the Eastern Red Sea deep-sea sediment; Table S3: Comparison of parameters between sediment layers (0–1 cm and 4–5 cm below seafloor) based on two-tailed Welch’s *t*-test; Table S4: Comparison of phylum-level dominant prokaryotes in deep-sea surface sediments between the upper OMZ-range and the below-OMZ based on two-tailed Welch’s *t*-test; Table S5: Depth-discriminatory bacterial and archaeal taxa in deep-sea surface sediments between the upper OMZ-range and the below-OMZ; Table S6: Summary of topological properties for prokaryotic co-occurrence networks at the phylum-, class-, and order levels; Table S7: Keystone taxa identified in prokaryotic co-occurrence networks at the phylum-, class-, and order levels; Table S8: Summary of topological properties for bacterial and archaeal co-occurrence networks at the phylum-, class-, and order levels; Figure S1: Latitudinal section of dissolved oxygen in the water column across the sediment-sampling stations along the Eastern Red Sea; Figure S2: Rank–abundance and cumulative rank–abundance curves of bacterial dOTUs; Figure S3: Rank–abundance and cumulative rank–abundance curves of archaeal dOTUs; Figure S4: Seafloor depth- and latitude-resolved patterns in Shannon diversity for prokaryotic communities in the deep-sea surface sediments along the Eastern Red Sea; Figure S5: NMDS ordination of archaeal communities in the deep-sea surface sediments along the Eastern Red Sea; Figure S6: Family-level depth-discriminatory archaeal taxa from deep-sea surface sediments differed between the upper OMZ-range and the below-OMZ; Figure S7: Co-occurrence networks of depth-discriminatory bacterial and archaeal taxa at the phylum level in deep-sea surface sediments in the upper OMZ-range and the below-OMZ; Figure S8: Co-occurrence networks of depth-discriminatory bacterial and archaeal taxa at the class level in deep-sea surface sediments in the upper OMZ-range and the below-OMZ; Figure S9: Co-occurrence networks of depth-discriminatory bacterial and archaeal taxa at the order level in deep-sea surface sediments in the upper OMZ-range and the below-OMZ.

## Abbreviations

The following abbreviations are used in this manuscript:

dOTUs	Denoised OTUs
PA	Prokaryotic abundance
PB	Prokaryotic biomass
POC	Particulate organic carbon
BP	Bacterial production
DO	Dissolved oxygen
OMZ	Oxygen minimum zone
SGR	Specific growth rate
