# Supplementary material for: Oxygen Minimum Zone and Organic Carbon Structure Benthic Prokaryotic Communities and Metabolism in Warm Deep-Red Sea Sediments
Source: Microorganisms. 2026 May 25;14(6):1191. doi: 10.3390/microorganisms14061191 (PMC13302808; doi:10.3390/microorganisms14061191)
Supplement: Supplementary file 1 [file microorganisms-14-01191-s001.zip › microorganisms-4287367-supplementary.pdf]

Table S1. Seafloor conditions along the Eastern Red Sea (DO: dissolved oxygen; ND: not detected).

Date	Station	Part	Seafloor depth	Latitude (° ND)	Longitude (° E)	Depth of CTD on ROV (m)	Temperature (°C)	bottom-water DO (μmol O_2 L^{-1})
15-Mar	L2P1D8	South	>500 m	16.56	41.04	1881	21.94	86.0
14-Mar	L2P1D7	South	>500 m	16.87	41.08	827	21.70	85.6
25-Mar	L2P2D1	South	200–500 m	16.93	41.14	268	21.95	69.4
26-Mar	L2P2D2	South	200–500 m	17.10	41.46	485	22.54	35.0
27-Mar	L2P2D3	South	200–500 m	17.20	40.97	298	21.81	57.3
17-Mar	L2P1D10	South	200–500 m	17.28	40.84	368	21.67	62.9
12-Mar	L2P1D5	South	>500 m	17.34	40.45	1050	21.75	94.2
13-Mar	L2P1D6	South	200–500 m	17.74	40.49	494	21.66	71.1
30-Mar	L2P2D6	South	200–500 m	17.82	40.71	217	22.09	75.6
18-Mar	L2P1D11	South	>500 m	17.89	40.37	862	21.71	85.2
10-Mar	L2P1D3	South	>500 m	18.26	39.88	1571	21.85	92.0
1-Apr	L2P2D8	South	200–500 m	18.51	40.66	301	21.88	53.0
11-Mar	L2P1D4	South	200–500 m	18.52	40.20	234	21.95	65.8
21-Mar	L2P1D14	South	>500 m	18.59	39.73	1693	21.88	94.1
20-Mar	L2P1D13	South	200–500 m	18.70	40.05	475	21.67	74.6
2-Apr	L2P2D9	South	200–500 m	18.81	40.64	302	21.84	43.5
4-Apr	L2P2D10	Center	200–500 m	19.09	40.37	286	ND	ND
8-Mar	L2P1D1	Center	>500 m	19.16	39.44	1311	21.80	99.2
9-Mar	L2P1D2	Center	200–500 m	19.22	39.61	502	21.70	61.0
12-Feb	L1P1D8	Center	>500 m	19.44	39.44	562	21.69	61.0
13-Feb	L1P1D9	Center	>500 m	19.63	38.98	1639	21.89	97.9
10-Feb	L1P1D6	Center	>500 m	20.01	38.63	1793	21.90	97.9
9-Feb	L1P1D5	Center	>500 m	20.67	38.97	680	21.71	74.1
8-Feb	L1P1D4	Center	>500 m	20.93	38.28	2408	22.07	96.1
7-Feb	L1P1D3	Center	>500 m	21.46	38.26	836	21.71	80.8
25-Apr	L3P1D1	Center	>500 m	22.41	38.68	701	21.72	73.8
1-May	L3P1D7	Center	>500 m	22.74	37.69	2023	21.96	97.8
26-Apr	L3P1D2	Center	>500 m	23.17	37.29	2263	22.03	97.3
30-Apr	L3P1D6	Center	200–500 m	23.64	37.88	396	21.69	57.1
27-Apr	L3P1D3	Center	>500 m	24.15	36.59	1581	21.90	97.6
29-Apr	L3P1D5	Center	>500 m	24.59	36.92	773	21.69	85.9
28-Apr	L3P1D4	Center	>500 m	24.73	36.27	1462	21.82	95.3
24-May	L4P1D1	North	>500 m	25.37	36.13	2225	22.02	109.3
27-May	L4P1D4	North	>500 m	26.58	35.89	893	21.75	95.8
26-May	L4P1D3	North	>500 m	26.79	35.21	945	21.73	126.4
28-May	L4P1D5	North	>500 m	27.71	34.62	1035	21.76	169.8
31-May	L4P1D8	North	>500 m	28.34	34.68	1001	21.26	185.6
30-May	L4P1D7	North	>500 m	28.79	34.80	1767	21.38	185.7
29-May	L4P1D6	North	>500 m	29.17	34.87	754	21.34	218.4

Table S2. Seafloor conditions and sediment microbial metrics across the Eastern Red Sea deep-sea sediments (POC: particulate organic carbon; PA: prokaryotic abundance; PB: prokaryotic biomass; PGA: prokaryotic [^{13}C]-D-glucose assimilation into biomass; SAR: biomass-specific glucose assimilation rate; ND: undetected).

Station	Depth of sediment cores (m)	Natural $\delta^{13}C$ of prokaryotic fraction (‰)		Sediment POC (%)		PA (× 10^{11} cells m^{-2})		PB (× 10^{-3} g C m^{-2})		PGA (mg C m^{-2} d^{-1})		SAR (× 10^{-3} d^{-1})	
		0–1 cm	4–5 cm	0–1 cm	4–5 cm	0–1 cm	4–5 cm	0–1 cm	4–5 cm	0–1 cm	4–5 cm	0–1 cm	4–5 cm
L2P1D8	1887	ND	ND	1.84	1.46	1.29	0.86	2.84	1.89	ND	ND	ND	ND
L2P1D7	826	–44.31	–45.86	1.54	1.62	9.11	6.03	20.03	13.26	0.11	0.11	5.29	8.40
L2P2D1	269	–32.28	–39.26	1.36	1.57	4.17	1.05	9.17	2.30	0.21	0.19	22.83	80.87
L2P2D2	486	–36.9	–33.01	1.44	1.62	6.30	3.62	13.87	7.96	0.22	0.20	15.89	25.52
L2P2D3	299	–54.08	–58.49	1.72	1.08	1.77	1.19	3.89	2.62	0.10	0.08	26.73	30.88
L2P1D10	368	ND	–53.04	1.70	1.51	1.13	1.93	2.49	4.25	0.02	0.10	7.10	24.07
L2P1D5	1053	ND	ND	1.71	1.67	1.76	0.52	3.86	1.15	ND	0.18	ND	158.99
L2P1D6	496	–55.72	–46.15	1.92	1.81	2.13	3.25	4.68	7.14	0.13	0.13	26.85	17.80
L2P2D6	217	ND	ND	1.99	1.70	2.45	1.81	5.39	3.98	0.03	0.11	5.32	28.53
L2P1D11	864	ND	ND	1.54	1.15	0.99	0.64	2.18	1.40	0.18	0.08	82.57	60.58
L2P1D3	1576	ND	ND	1.36	1.70	2.44	1.20	5.37	2.64	ND	ND	ND	ND
L2P2D8	302	–41.58	–41.29	1.89	1.75	2.36	1.91	5.19	4.20	0.04	0.08	7.64	18.93
L2P1D4	235	ND	ND	1.27	1.54	1.52	4.00	3.33	8.80	0.15	0.02	44.57	1.91
L2P1D14	1697	ND	ND	1.43	0.91	0.61	0.31	1.34	0.68	ND	ND	ND	ND
L2P1D13	476	–51.83	–43.38	1.72	1.61	3.80	4.98	8.37	10.95	0.11	0.12	12.75	11.02
L2P2D9	303	–48.03	–39.17	1.12	1.26	2.44	2.70	5.38	5.94	0.10	0.11	17.81	18.39
L2P2D10	286	–44.88	–51.52	1.60	1.52	9.91	4.21	21.79	9.26	0.06	0.11	2.66	11.40
L2P1D1	1315	ND	ND	1.11	1.42	1.99	0.63	4.38	1.39	ND	ND	ND	ND
L2P1D2	503	ND	ND	1.54	1.55	4.94	4.28	10.86	9.43	ND	ND	ND	ND
L1P1D8	563	ND	ND	1.05	1.19	2.06	0.47	4.52	1.04	ND	ND	ND	ND
L1P1D9	1644	ND	ND	1.00	1.41	1.21	0.62	2.67	1.37	ND	ND	ND	ND
L1P1D6	1798	ND	ND	1.11	1.12	2.16	3.59	4.74	7.90	ND	ND	ND	ND
L1P1D5	682	ND	ND	0.94	1.24	5.38	1.31	11.83	2.89	ND	ND	ND	ND
L1P1D4	2415	ND	ND	1.44	0.95	0.94	0.28	2.07	0.61	ND	ND	ND	ND
L1P1D3	839	ND	ND	1.07	0.70	0.45	0.49	0.98	1.07	ND	ND	ND	ND
L3P1D1	703	ND	ND	1.12	0.56	1.46	0.27	3.22	0.60	0.11	ND	35.63	ND
L3P1D7	2029	ND	ND	0.83	0.58	0.61	0.50	1.34	1.11	ND	ND	ND	ND
L3P1D2	2270	ND	ND	0.57	0.58	0.55	0.20	1.22	0.44	ND	ND	ND	ND
L3P1D6	397	ND	ND	0.39	0.44	0.60	0.50	1.32	1.10	ND	ND	ND	ND
L3P1D3	1585	ND	ND	0.44	0.49	0.37	0.47	0.82	1.04	0.13	0.06	162.28	55.18
L3P1D5	775	ND	ND	0.77	0.34	0.51	0.37	1.13	0.82	ND	ND	ND	ND
L3P1D4	1469	ND	ND	0.45	0.77	3.46	0.62	7.62	1.36	ND	ND	ND	ND
L4P1D1	2258	ND	ND	0.45	0.42	0.62	0.15	1.37	0.34	0.05	ND	33.04	ND
L4P1D4	895	ND	ND	0.33	0.39	0.46	0.19	1.00	0.42	ND	ND	ND	ND
L4P1D3	947	ND	ND	0.37	0.33	0.36	0.54	0.79	1.19	ND	ND	ND	ND
L4P1D5	1038	ND	ND	0.32	0.33	1.19	0.77	2.62	1.68	0.07	0.03	25.60	20.21
L4P1D8	1001	ND	ND	0.50	0.28	0.23	0.48	0.50	1.06	ND	ND	ND	ND
L4P1D7	1772	–56.97	–59.96	0.62	0.73	0.89	0.64	1.95	1.41	0.02	0.01	8.73	9.83
L4P1D6	756	ND	ND	0.33	0.32	0.60	0.36	1.32	0.78	ND	ND	ND	ND

Table S3. Comparison of parameters between sediment layers (0–1 cm and 4–5 cm below seafloor) based on two-tailed Welch's t-test (POC: particulate organic carbon; PA: prokaryotic abundance; PB: prokaryotic biomass; PGA: prokaryotic [^{13}C]-D-glucose assimilation into biomass; SAR: biomass-specific glucose assimilation rate).

**Parameters**	**Sediment layer**	***n***	**Mean ± SD**	***t***	**df**	***p***
Sediment POC (%)	0–1 cm	39	1.13 ± 0.53	0.490	75.97	0.625
	4–5 cm	39	1.07 ± 0.52			
Natural $\delta^{13}C$ of prokaryotic fraction (‰)	0–1 cm	11	–47.1 ± 7.94	–0.181	19.92	0.858
	4–5 cm	11	–46.47 ± 8.47			
PA (cells m^{-2})	0–1 cm	39	$(2.18 \pm 2.26) \times 10^{11}$	1.585	67.58	0.118
	4–5 cm	39	$(1.49 \pm 1.57) \times 10^{11}$			
PB (g C m^{-2})	0–1 cm	39	$(4.81 \pm 4.98) \times 10^{3}$	1.585	67.58	0.118
	4–5 cm	39	$(3.27 \pm 3.44) \times 10^{3}$			
PGA (mg C m^{-2} d^{-1})	0–1 cm	18	0.10 ± 0.06	–0.041	32.86	0.968
	4–5 cm	17	0.10 ± 0.06			
SAR (d^{-1})	0–1 cm	18	$(30.18 \pm 38.12) \times 10^{-3}$	–0.316	32.87	0.754
	4–5 cm	17	$(34.27 \pm 38.26) \times 10^{-3}$			

Table S4. Comparison of phylum-level dominant prokaryotes in deep-sea surface sediments (0–1 cm below seafloor) between two seafloor-depth groups (200–500 m: upper OMZ-range; >500 m: below-OMZ) based on two-tailed Welch's *t*-test. Statistically significant differences ($p < 0.05$) are shown in bold.

Domain	Phylum	Seafloor depth	*n*	*t*	df	*p*
Bacteria	Pseudomonadota	200–500 m	13	1.538	24.25	0.137
		>500 m	19			
Bacteria	Acidobacteriota	200–500 m	12	2.701	19.44	**< 0.05**
		>500 m	18			
Bacteria	Planctomycetota	200–500 m	12	1.076	26.02	0.292
		>500 m	18			
Bacteria	Chloroflexota	200–500 m	12	−2.218	15.68	**< 0.05**
		>500 m	18			
Bacteria	Actinomycetota	200–500 m	12	3.129	19.83	**< 0.01**
		>500 m	18			
Bacteria	NB1-j	200–500 m	12	1.889	20.46	0.073
		>500 m	18			
Bacteria	Gemmatimonadota	200–500 m	12	2.754	20.15	**< 0.05**
		>500 m	18			
Bacteria	Methylomirabilota	200–500 m	12	3.503	26.19	**< 0.01**
		>500 m	18			
Bacteria	Nitrospirota	200–500 m	12	0.111	22.42	0.913
		>500 m	18			
Bacteria	Desulfobacterota	200–500 m	12	−2.842	14.93	**< 0.05**
		>500 m	18			
Bacteria	Verrucomicrobiota	200–500 m	12	−0.074	27.99	0.942
		>500 m	18			
Bacteria	Myxococcota	200–500 m	12	0.956	26.19	0.348
		>500 m	18			
Bacteria	Patescibacteria	200–500 m	12	−0.018	21.08	0.985
		>500 m	18			
Archaea	Thermoplasmatota	200–500 m	12	1.622	19.87	0.121
		>500 m	18			
Archaea	Nanobdellota	200–500 m	12	0.650	27.46	0.521
		>500 m	18			
Archaea	Thermoproteota	200–500 m	12	0.731	21.28	0.473
		>500 m	18			
Archaea	Aenigmarchaeota	200–500 m	12	−1.625	11.27	0.132
		>500 m	18			
Archaea	Methanobacteriota	200–500 m	12	−1.568	11.30	0.144
		>500 m	18			
Archaea	Halobacteriota	200–500 m	12	−2.340	15.87	**< 0.05**
		>500 m	18			
Archaea	Asgardarchaeota	200–500 m	12	−2.743	13.83	**< 0.05**
		>500 m	18			
Archaea	Iainarchaeota	200–500 m	12	−1.867	11.27	0.088
		>500 m	18			
Archaea	Micrarchaeota	200–500 m	12	−1.452	14.03	0.169
		>500 m	18			
Archaea	Hadarchaeota	200–500 m	12	−1.402	11.00	0.189
		>500 m	18			
Archaea	Altiarchaeota	200–500 m	12	−1.724	11.09	0.112
		>500 m	18			

Table S5. Depth-discriminatory bacterial and archaeal taxa in deep-sea surface sediments (0–1 cm below seafloor) between two seafloor-depth groups (200–500 m: upper OMZ-range; >500 m: below-OMZ). Differences in relative abundance were tested using two-sided Mann–Whitney U tests. W denotes the test statistics. *p*-values indicate unadjusted significance levels, and *q*-values represent Benjamini–Hochberg FDR–corrected *p*-values.

Domai	Taxonom	Taxon	Numbe	Number	Number	W (Mann–	*p*	*q*
Bacteri	Family	Anaerolineaceae	32	13	19	198	4.52×10^{-3}	5.27×10^{-3}
Bacteri	Family	Desulfatiglandaceae	32	13	19	231	4.03×10^{-5}	1.41×10^{-4}
Bacteri	Family	Desulfosarcinaceae	32	13	19	235	2.05×10^{-5}	1.41×10^{-4}
Bacteri	Family	Gemmatimonadaceae	32	13	19	66	0.029	0.029
Bacteri	Family	Hyphomicrobiaceae	32	13	19	200	3.48×10^{-3}	4.88×10^{-3}
Bacteri	Family	Sandaracinaceae	32	13	19	211	8.43×10^{-4}	1.48×10^{-3}
Bacteri	Family	Spirochaetaceae	32	13	19	227	7.75×10^{-5}	1.81×10^{-4}
Bacteri	Genus	*Desulfatiglans*	32	13	19	231	4.02×10^{-5}	9.38×10^{-5}
Bacteri	Genus	Pir4 lineage	32	13	19	184	0.021	0.021
Bacteri	Genus	SEEP-SRB1	32	13	19	220	2.28×10^{-4}	2.66×10^{-4}
Bacteri	Genus	*Spirochaeta*	32	13	19	223	1.45×10^{-4}	2.03×10^{-4}
Bacteri	Genus	Subgroup 23	32	13	19	231	4.01×10^{-5}	9.38×10^{-5}
Bacteri	Genus	Sva0081 sediment	32	13	19	237	1.44×10^{-5}	9.38×10^{-5}
Bacteri	Genus	*Thermoflexus*	32	13	19	223	1.41×10^{-4}	2.03×10^{-4}
Archae	Family	Caldiarchaeaceae	32	13	19	161.5	0.012	0.020
Archae	Family	CG1–02–57–44	32	13	19	182.5	0.019	0.024
Archae	Family	Geothermarchaeaceae	32	13	19	175.5	0.046	0.046
Archae	Family	SCGC_AAA011-D5	32	13	19	200	0.003	0.017
Archae	Family	Syntrophoarchaeacea	32	13	19	161.5	0.012	0.020

Table S6. Summary of topological properties for prokaryotic co-occurrence networks (200–500 m: upper OMZ-range vs >500 m: below-OMZ) at the phylum-, class-, and order levels (OMZ: oxygen minimum zone).

Taxonomic level	Seafloor depth	Nodes	Edges	Positive/Negative edges	Average degree	Connected components	Clustering Coefficient
Phylum	200–500 m	13	33	15/18	5.08	1	0.754
Phylum	>500 m	13	18	12/6	2.77	2	0.491
Class	200–500 m	17	56	24/32	6.59	1	0.637
Class	>500 m	19	47	31/16	4.95	1	0.744
Order	200–500 m	16	29	17/12	3.63	2	0.559
Order	>500 m	19	34	25/9	3.58	3	0.545

Table S7. Keystone taxa identified in prokaryotic co-occurrence networks (200–500 m: upper OMZ-range vs >500 m: below-OMZ) at the phylum-, class-, and order levels based on the intersection of the top 20% nodes ranked by degree and betweenness centrality. MBG-D and DHVEG-1: Marine Benthic Group D and Deep-sea Hydrothermal Vent Methanobacterial Group 1. OMZ: oxygen minimum zone.

Taxonomic level	Seafloor depth	Domain	Keystone taxon	Node size	Degree	Betweenness centrality
Phylum	200–500 m	Bacteria	Spirochaetota	0.016	8	0.333
Phylum	>500 m	Bacteria	Chloroflexota	0.202	5	0.258
Phylum	>500 m	Archaea	Hydrothermarchaeota	0.136	7	0.364
Class	200–500 m	Bacteria	Vicinamibacteria	0.062	12	0.133
Class	200–500 m	Archaea	Nitrososphaeria	0.221	12	0.168
Order	200–500 m	Bacteria	Rokubacteriales	0.039	7	0.221
Order	200–500 m	Bacteria	SAR202_clade	0.030	6	0.141
Order	200–500 m	Archaea	MBG-D and DHVEG-1	0.290	8	0.207
Order	>500 m	Bacteria	Aminicenantales	0.043	7	0.120
Order	>500 m	Archaea	MBG-D and DHVEG-1	0.009	9	0.191
Order	>500 m	Archaea	Hydrothermarchaeales	0.072	9	0.138

Table S8. Summary of topological properties for bacterial and archaeal co-occurrence networks (200–500 m: upper OMZ-range vs >500 m: below-OMZ) at the phylum-, class-, and order levels (OMZ: oxygen minimum zone; ND: not detected).

Domain	Taxonomic [illegible]	Seafloor [illegible]	Nodes	Edges	Positive/Ne[illegible]	Average [illegible]	Connected [illegible]	Clustering [illegible]
Bacteria	Phylum	200–500 m	11	20	9/11	3.64	2	0.760
Bacteria	Phylum	>500 m	11	10	7/3	1.82	3	0.353
Archaea	Phylum	200–500 m	3	1	0/1	0.67	2	ND
Archaea	Phylum	>500 m	3	2	0/2	1.33	1	0.000
Bacteria	Class	200–500 m	11	20	11/9	3.64	2	0.671
Bacteria	Class	>500 m	11	11	7/4	2.00	3	0.529
Archaea	Class	200–500 m	9	10	4/6	2.22	4	0.667
Archaea	Class	>500 m	9	13	7/6	2.89	2	0.698
Bacteria	Order	200–500 m	11	14	9/5	2.55	3	0.405
Bacteria	Order	>500 m	11	11	8/3	2.00	3	0.474
Archaea	Order	200–500 m	10	0	0/0	0.00	10	ND
Archaea	Order	>500 m	10	6	5/1	1.20	4	0.000

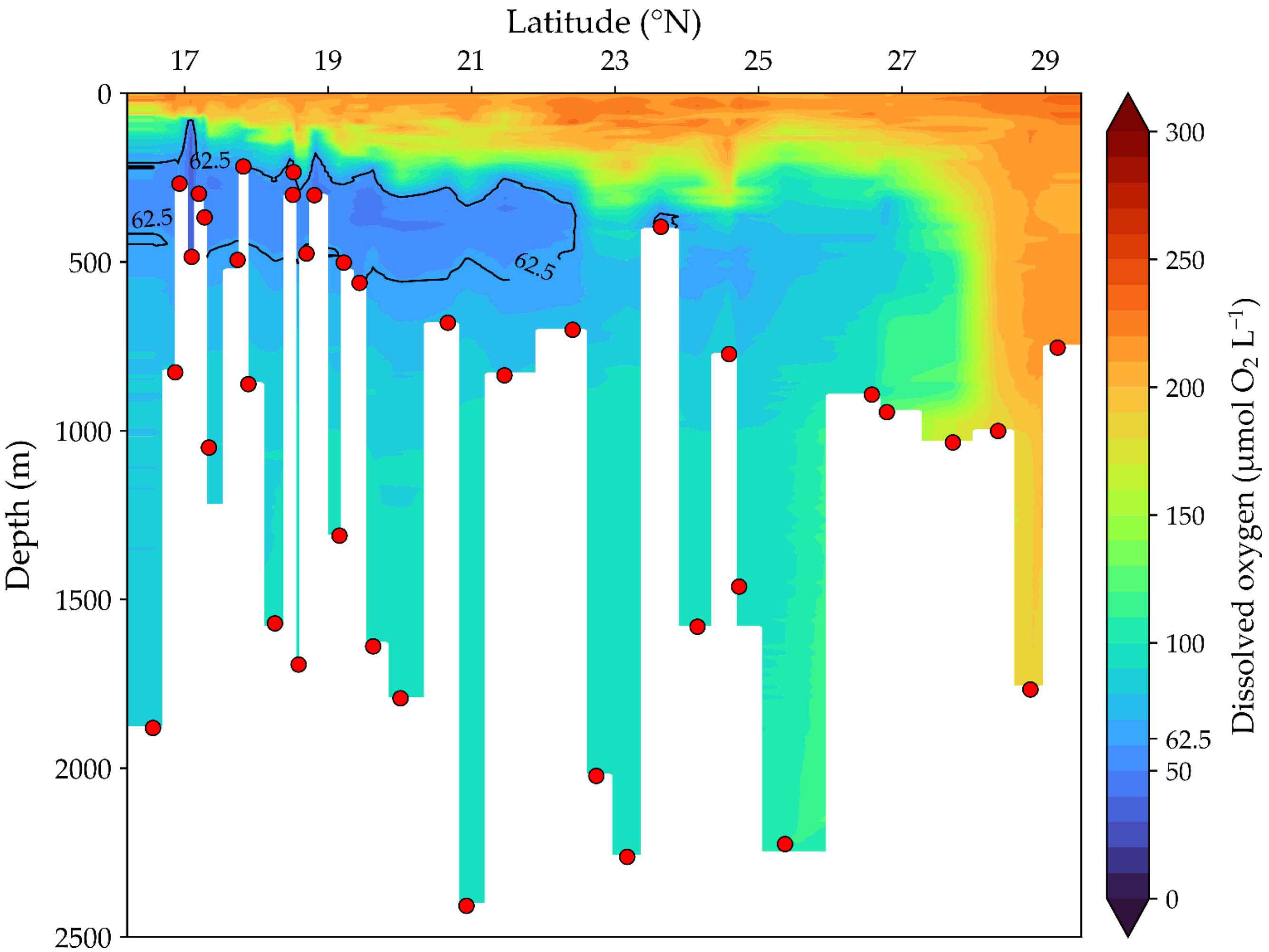

Figure S1. Latitudinal section of dissolved oxygen (DO, µmol O_2 L^{-1}) in the water column across the sediment-sampling stations along the Eastern Red Sea. Red dots indicate sediment-sampling stations at their corresponding latitude and seafloor depth. The black contour line marks the 62.5 µmol O_2 L^{-1} reference hypoxia threshold.

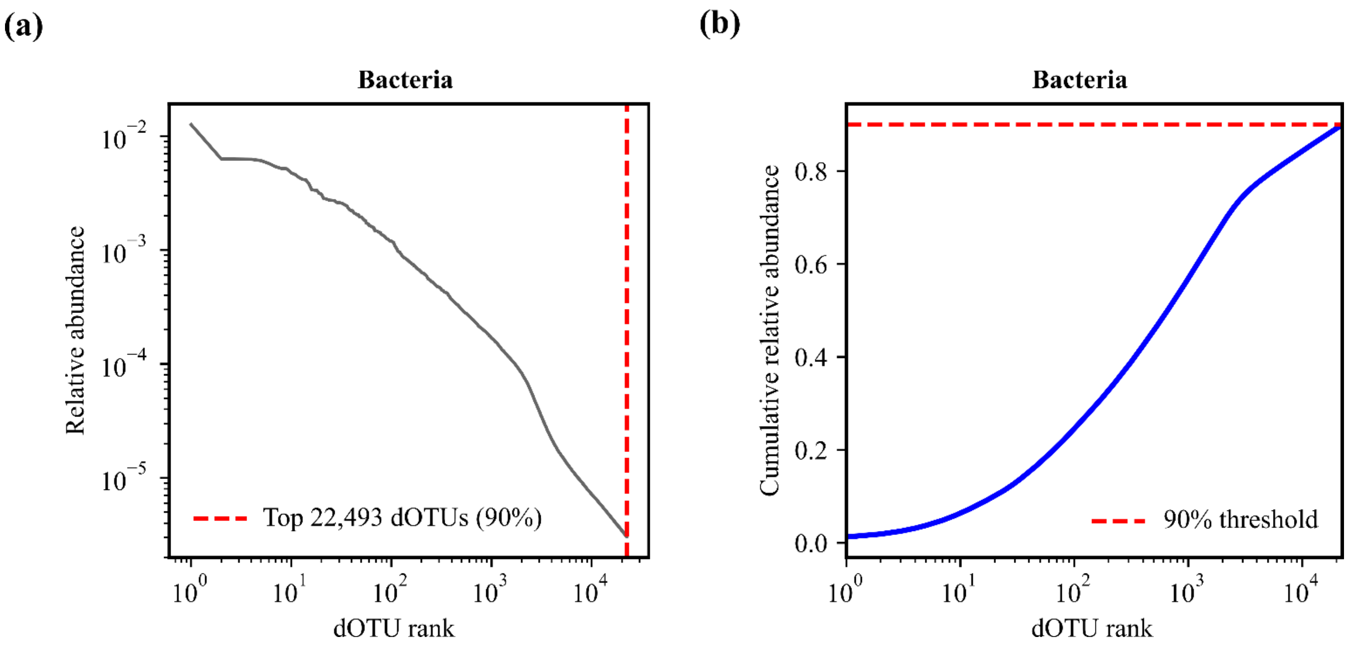

Figure S2. Rank–abundance and cumulative rank–abundance curves of bacterial dOTUs (denoised OTUs). **(a)** Rank–abundance curve (log–log scale) showing the relative abundance of dOTUs ordered from most to least abundant **(b)** Cumulative relative abundance curve. The dashed red lines indicate the threshold where highest-abundance subset of dOTUs cumulatively accounts for 90% of the total community relative abundance.

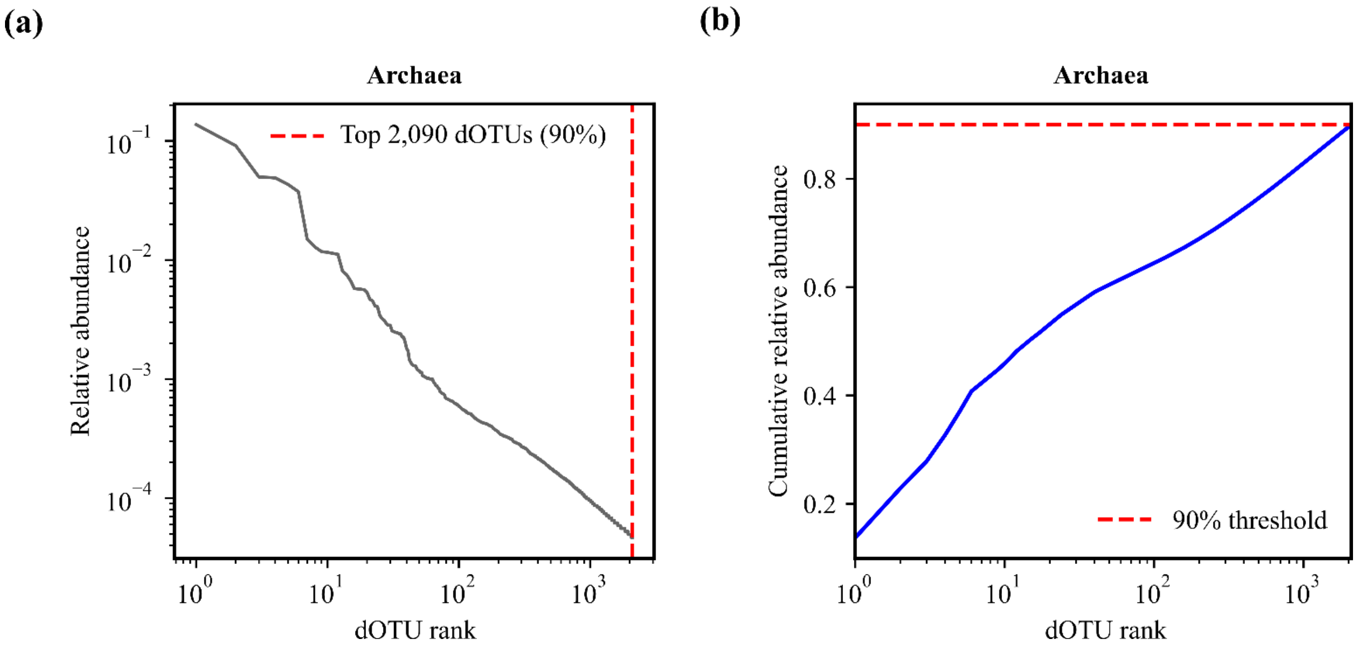

Figure S3. Rank–abundance and cumulative rank–abundance curves of archaeal dOTUs (denoised OTUs). (**a**) Rank–abundance curve (log–log scale) showing the relative abundance of dOTUs ordered from most to least abundant. (**b**) Cumulative relative abundance curve. The dashed red lines indicate the threshold where the highest-abundance subset of dOTUs cumulatively accounts for 90% of the total community relative abundance.

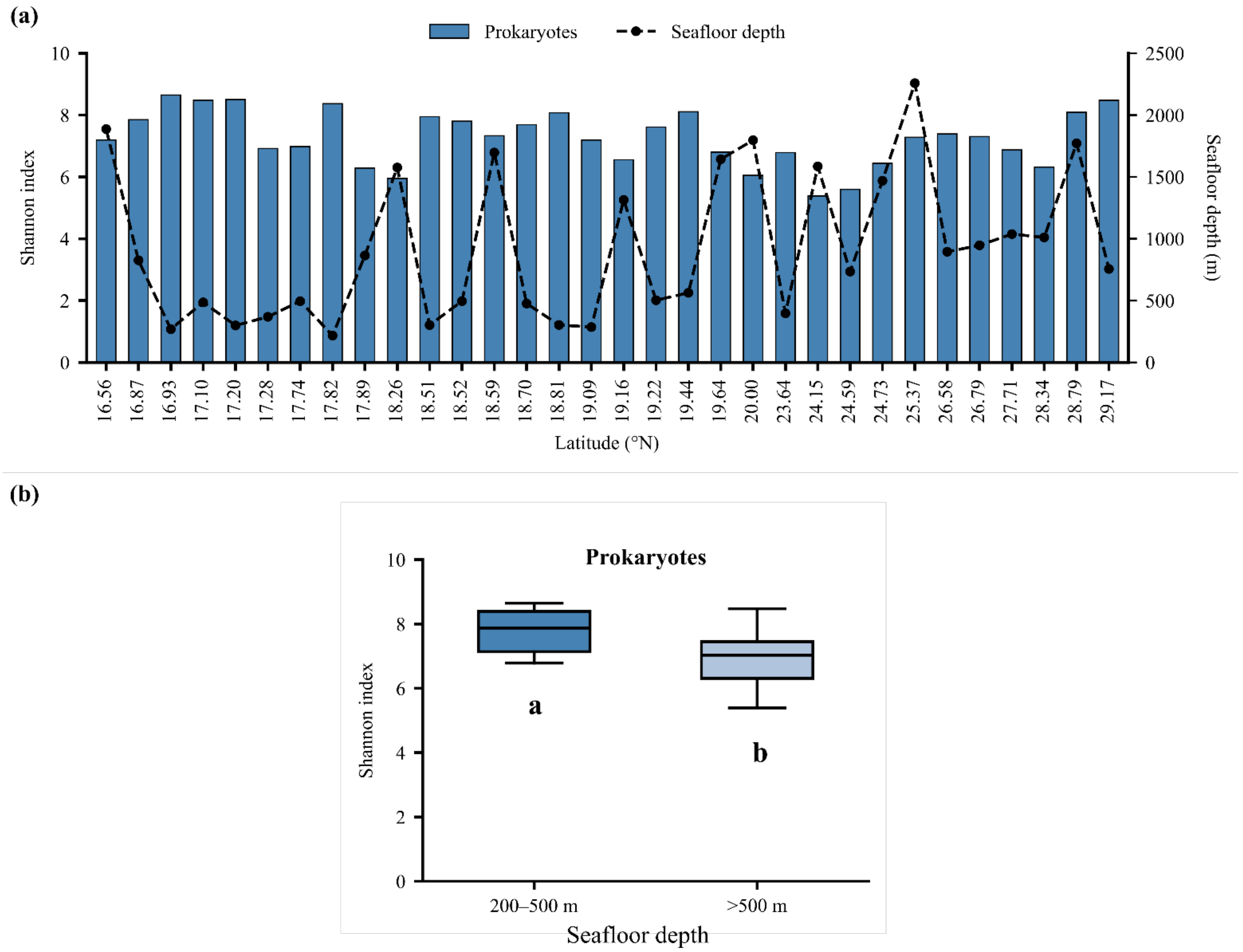

Figure S4. Seafloor depth- and latitude-resolved patterns in Shannon diversity for prokaryotic communities in the deep-sea surface sediments (0–1 cm below seafloor) along the Eastern Red Sea. (**a**) Shannon index for the combined domain across stations ordered by latitude (south → north), with seafloor depth overlaid as a dashed line. (**b**) Boxplots comparing Shannon diversity between the upper OMZ-range (200–500 m seafloor depth) and the below-OMZ (>500 m). Boxes that do not share the same letter showed significant differences ($p < 0.05$).

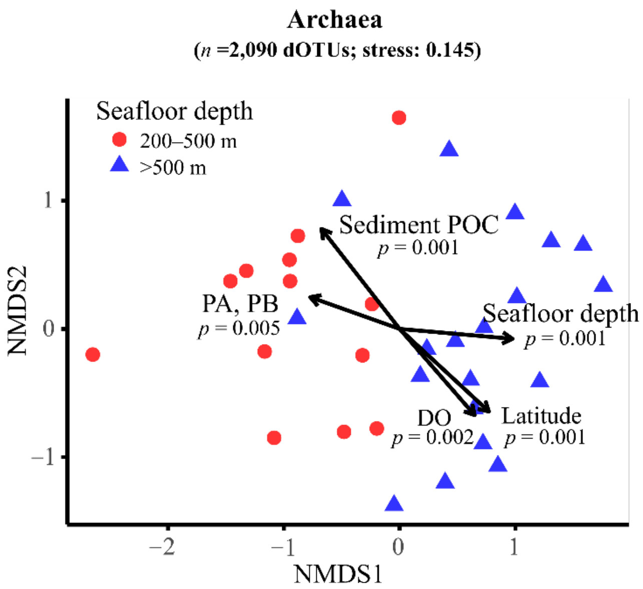

Figure S5. NMDS ordination (Bray–Curtis) of archaeal communities in the deep-sea surface sediments (0–1 cm below seafloor) along the Eastern Red Sea, based on the subset of dOTUs (denoised OTUs) cumulatively accounting for 90% of the total relative abundance, with benthic samples divided by their relation to the oxygen minimum zone (OMZ) (upper OMZ-range, red circles; below-OMZ, blue triangles). Black vectors show significant environmental fits ($p < 0.05$), including seafloor depth, bottom-water dissolved oxygen (DO), latitude, sediment particulate organic carbon (POC), prokaryotic abundance (PA), and prokaryotic biomass (PB); vector direction indicates increasing values and vector length reflects the strength of correlation with the ordination.

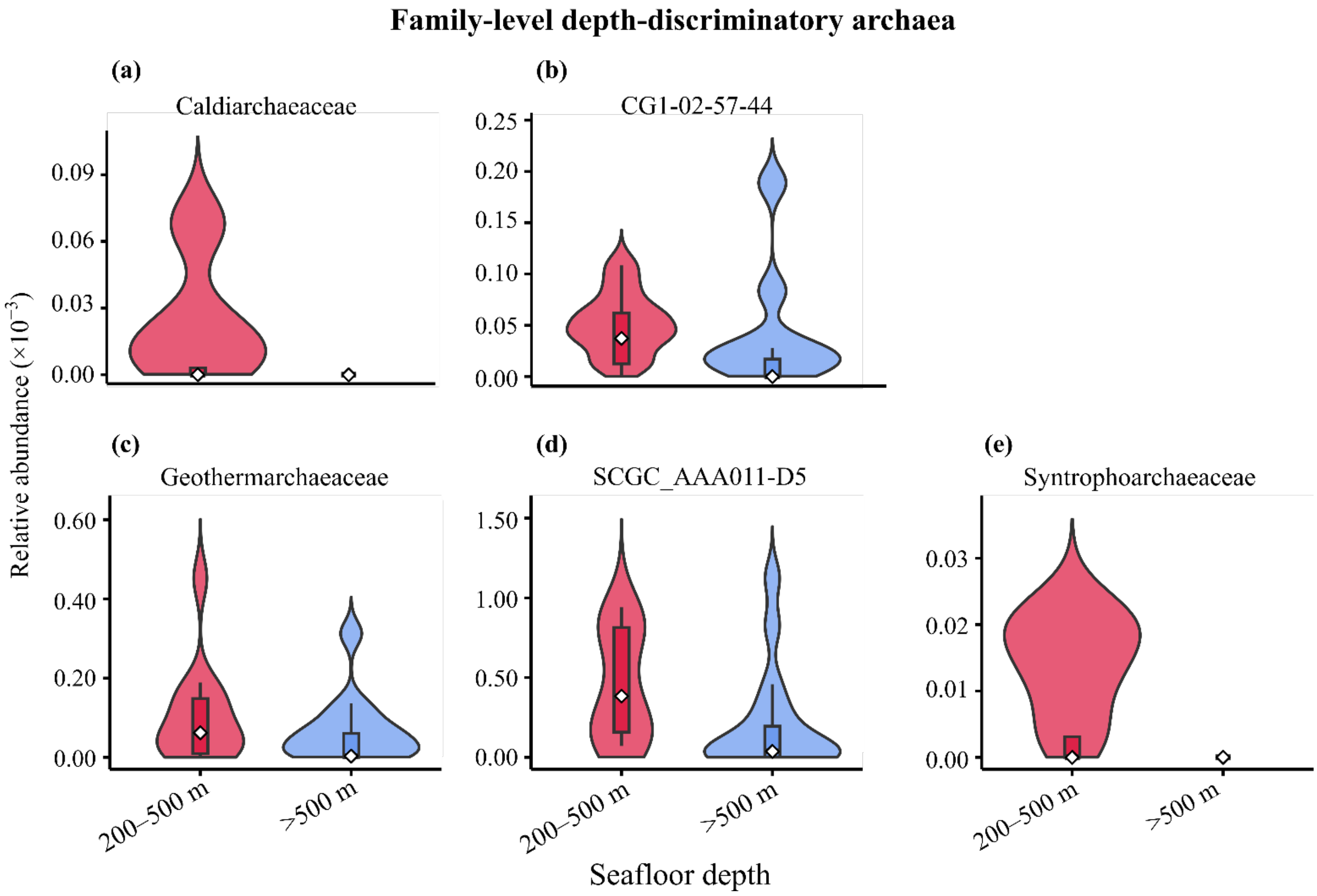

Figure S6. Family-level violin plots representing the relative reads of depth-discriminatory archaeal taxa from deep-sea surface sediments (0–1 cm below seafloor) between the upper OMZ-range (200–500 m: red) and the below-OMZ (>500 m; blue). (**a**) Caldiarchaeaceae. (**b**) CG1–02–57–44. (**c**) Geothermarchaeaceae. (**d**) SCGC_AAA011-D5. (**e**) Syntrophoarchaeaceae. Depth-discriminatory taxa were identified using a two-step selection: (i) taxa that differed significantly in relative abundance between the two zones (two-sided Mann–Whitney U test with Benjamini–Hochberg FDR-adjusted $q < 0.05$), and (ii) taxa ranked among the top 20 contributors to between-zone Bray–Curtis dissimilarity based on SIMPER analysis. The final set comprised taxa meeting both criteria. For clarity, only the upper half of each violin is shown.

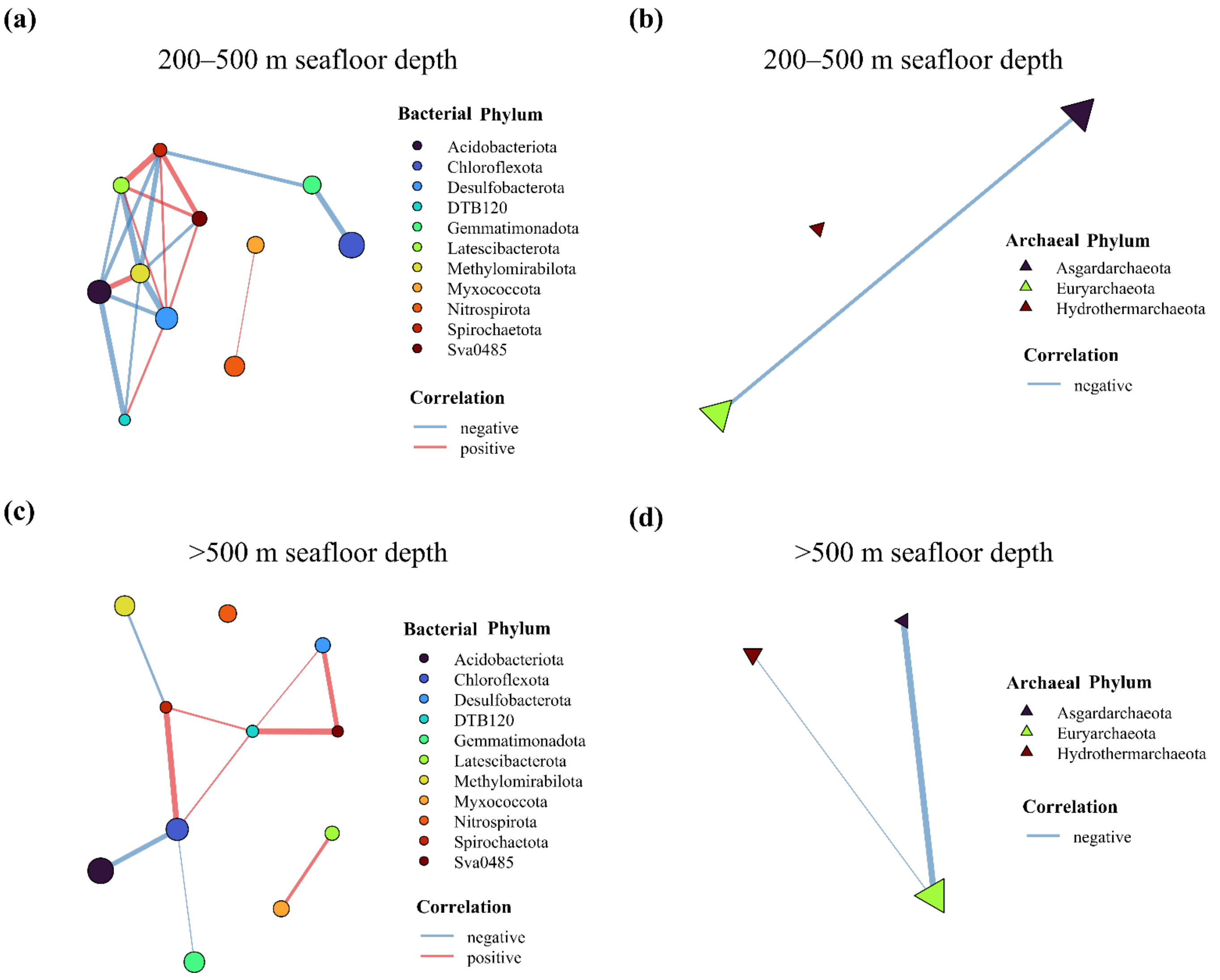

Figure S7. Co-occurrence networks of depth-discriminatory bacterial and archaeal taxa at the phylum level in deep-sea surface sediments (0–1 cm below seafloor) constructed from Spearman correlations of relative abundances. (**a**, **b**) represent communities at 200–500 m, and (**c**, **d**) at > 500 m seafloor depths. Depth-discriminatory taxa were identified using a two-step selection: (i) taxa that differed significantly in relative abundance between the two zones (two-sided Mann–Whitney U test with Benjamini–Hochberg FDR-adjusted $q < 0.05$), and (ii) taxa ranked among the top 20 contributors to between-zone Bray–Curtis dissimilarity based on SIMPER analysis. The final set comprised taxa meeting both criteria. Edges were retained when $|\rho| \geqslant 0.6$ and Benjamini – Hochberg FDR-adjusted $q < 0.05$; red edges indicate positive correlations and blue edges indicate negative correlations, with edge width proportional to $|\rho|$. Nodes represent depth-discriminatory taxa; node size is proportional to mean relative abundance within each zone, and node color distinguishes individual taxa.

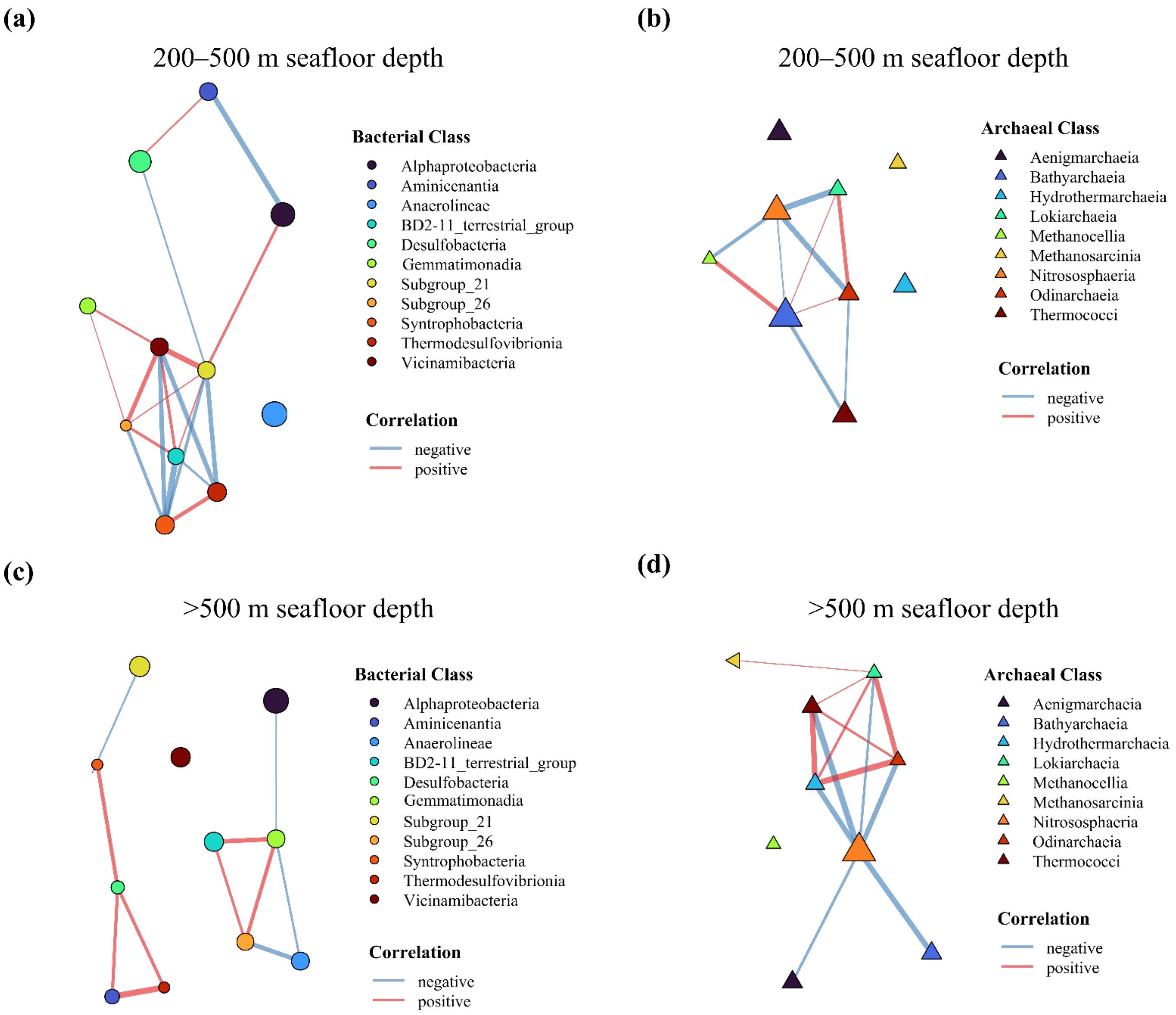

Figure S8. Co-occurrence networks of depth-discriminatory bacterial and archaeal taxa at the class level in deep-sea surface sediments (0–1 cm below seafloor) constructed from Spearman correlations of relative abundances. (**a**, **b**) represent communities at 200–500 m, and (**c**, **d**) at > 500 m seafloor depths. Selection criteria as described in **Figure S7**. Edges and nodes as described in **Figure S7**.

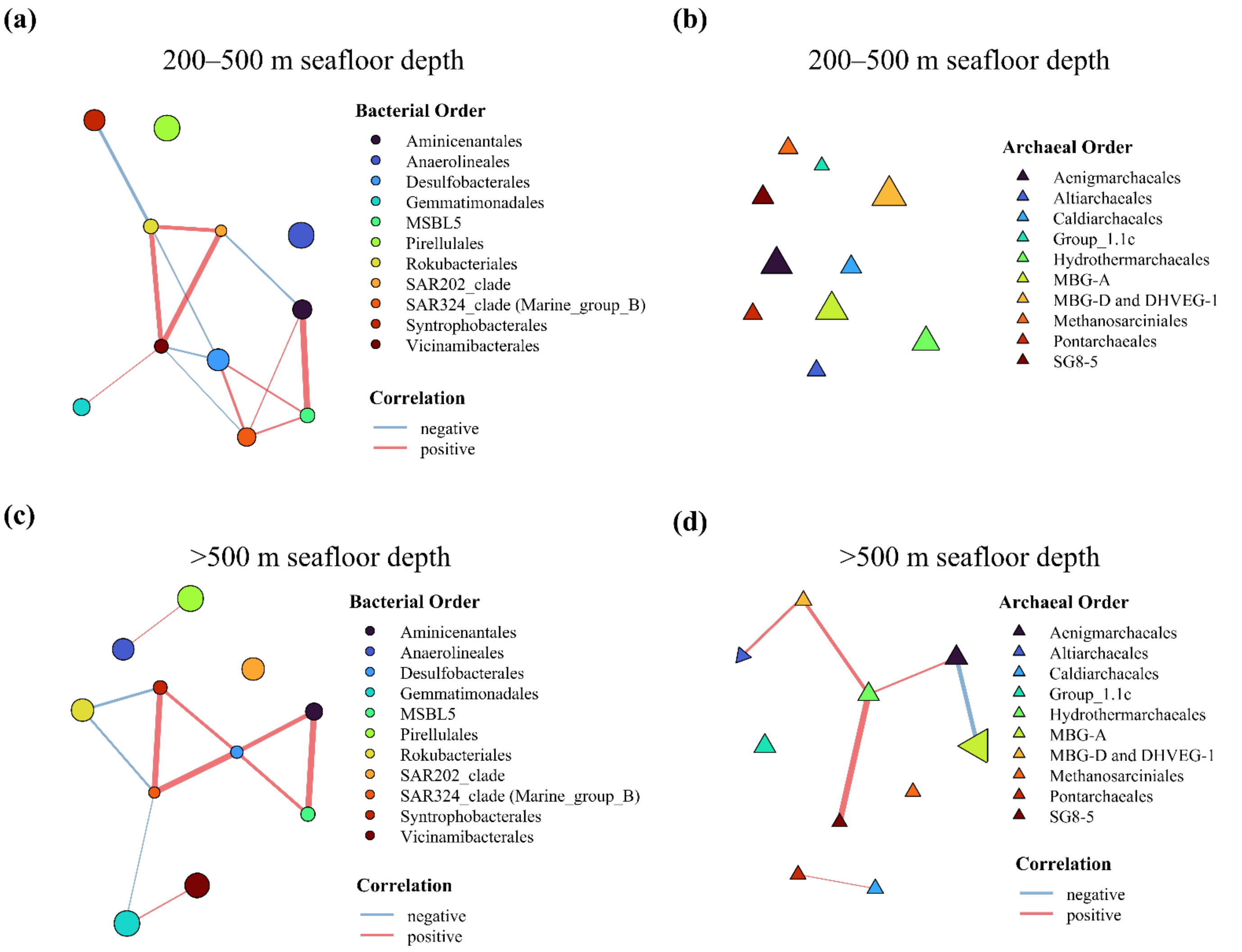

Figure S9. Co-occurrence networks of depth-discriminatory bacterial and archaeal taxa at the order level in deep-sea surface sediments (0–1 cm below seafloor) constructed from Spearman correlations of relative abundances. (**a**, **b**) represent communities at 200–500 m, and (**c**, **d**) at > 500 m seafloor depths. Selection criteria as described in **Figures S7** and **S8**. Edges and nodes as described in **Figures S7** and **S8**.
